# Unveiling Anisotropic in‐Plane Thermal Conductivity and Thermal Expansion Coefficient in Low‐Symmetry Few‐Layer ReS_2_

**DOI:** 10.1002/advs.77734

**Published:** 2026-09-27

**Authors:** Manab Mandal, Prahalad Kanti Barman, Susmita Jana, Saroj Poudyal, Kaushalya Kumari, Sourav Mondal, Abhishek Misra, Birabar Ranjit Kumar Nanda, Pramoda Kumar Nayak, Kanikrishnan Sethupathi

**Affiliations:** ^1^ Low Temperature Physics Laboratory Department of Physics Indian Institute of Technology Madras Chennai India; ^2^ 2D Materials Research and Innovation Group Department of Physics Indian Institute of Technology Madras Chennai India; ^3^ Center for Atomistic Modelling and Materials Design Department of Physics Indian Institute of Technology Madras Chennai India; ^4^ Centre for Nano and Material Sciences Jain (Deemed‐to‐be University) Jain Global Campus Bangalore India

**Keywords:** anisotropic 2D materials, Raman thermometry, ReS_2_, thermal conductivity, thermal expansion coefficient

## Abstract

ReS_2_ belongs to a unique class of 2D materials with low structural symmetry and extremely weak interlayer bonding, which give rise to intriguing thermal properties, like in‐plane anisotropic thermal conductivity (ATC) and anisotropic thermal expansion coefficient (ATEC). This study provides experimental evidence of in‐plane ATC and ATEC in few‐layer ReS_2_, revealed through simultaneous two‐mode Raman thermometry. In a single optical configuration, ATC of ReS_2_ has been determined by simultaneous analysis of two anisotropic Raman modes (III and V) corresponding to in‐plane crystallographic axes. For the five‐layer (5L) ReS_2_, the extracted thermal conductivities are ∼ 28.78 (‘*b*’‐axis) and ∼ 22.25 (‘*a*’‐axis) Wm^−1^K^−1^, respectively while the corresponding TECs are ∼ 6.1 × 10^−6^ and ∼ 4.04 × 10^−6^ K^−1^. As the layer thickness varies from 5 to 8L, the ATC and ATEC ratios monotonically increase from 1.29 and 1.52 to 1.36 and 1.94, respectively. The ab initio calculations reveal that the directional in‐plane anisotropy in phonon dispersion, group velocity, and Grüneisen parameter gives rise to ATC and ATEC. This work paves the way for exploring in‐plane ATC and ATEC in other low‐symmetry 2D materials, with potential implications for device applications.

## Graphical abstract

Simultaneous two‐mode Raman thermometry reveals strong in‐plane anisotropic thermal conductivity and thermal expansion coefficients in low‐symmetry few‐layer ReS_2_. By decoupling thermal transport along the crystallographic *a*‐ and *b*‐axes within a single optical configuration, this study provides robust experimental evidence and ab initio theoretical insights into nanoscale directional heat flow, paving the way for advanced thermal management in 2D materials.

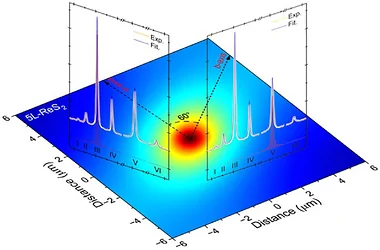

## 1 Introduction

Symmetry constitutes a crucial role in the fundamental laws of nature [1, 2, 3, 4], governing classical equations of motion, conservation laws, quantum selection rules, and exchange interactions [5, 6, 7]. In condensed matter, symmetry dictates a broad range of mechanical, electronic, and optical responses, including stress‐strain relations, charge transport, conductivity, refractive index, and the nature of allowed nonlinear processes [8, 9, 10]. Crystals with high structural symmetry typically exhibit isotropic electronic, optical, and thermal properties, which suppress phenomena such as even‐harmonic generation, chirality, and birefringence [3, 11, 12]. In contrast, lowering the crystal symmetry gives rise to anisotropy, where a given physical property becomes axial [4, 13]. The 2D Van der Waals (vdW) layered systems present an ideal platform for exploring such effects due to their intrinsic structural anisotropy arising from the strong intralayer covalent bonding and weak interlayer vdW interactions [14]. This anisotropy facilitates a variety of exotic light‐matter interactions, leading to exciton, phonon, edge, and moiré polaritons [15, 16, 17]. While the majority of 2D vdW materials exhibit isotropic behavior, certain systems with in‐plane low symmetry, such as ReX_2_ (X = S, Se), demonstrate biaxial anisotropy [18, 19, 20, 21, 22]. In these materials, the non‐orthogonal orientation of in‐plane excitonic and phononic resonances induces a wavelength‐ and frequency‐dependent rotation of the principal axes, further enriching their optical and vibrational characteristics [19, 23, 24, 25].

In this context, symmetry lowering and anisotropy not only influence optical processes but also play a crucial role in thermal transport. In‐plane structural anisotropy governs phonon dispersion, scattering mechanisms, thermal expansion, and heat conduction pathways, thereby giving rise to strongly direction‐dependent TC and TEC. Among other 2D TMDCs, the *1T'* stable phase of ReS_2_ is one of the promising candidates to exhibit strong thermal anisotropy. Although anisotropic optical and electrical properties of layered ReS_2_ have been extensively studied [26, 27, 28, 29] [30], systematic investigations of its thermal transport, particularly in the 2D limit, remain unexplored. In the bulk limit, ReS_2_ exhibits in‐plane thermal conductivities of (70±18) and (50±13) Wm^−1^K^−1^, corresponding to an anisotropy ratio of 1.4 [31]. Other low‐symmetry materials, like Td‐WTe_2_ and Black Phosphorus (BP), show notable in‐plane ATC. Td‐WTe_2_ exhibits ATCs of 0.743 and 0.639 Wm^−1^K^−1^ along the zigzag and armchair axes, respectively [32]. Luo et al. reported that, for BP films thicker than 15 nm, the armchair and zigzag thermal conductivities are ∼ 20 and ∼ 40 Wm^−1^K^−1^, respectively [33]. In previous studies, ATC has been determined by analyzing specific Raman modes (power‐ and temperature‐dependent evolution) along either the zigzag or armchair directions [32, 33]. However, in those studies, the laser polarization had to be precisely aligned with the zigzag and armchair orientations to accurately probe the ATC. In contrast, our measurement approach is simpler and fundamentally different. Here, we first identify the Raman modes associated with vibrations along the in‐plane crystallographic axes. Consequently, these modes exhibit anisotropic heat propagation along the respective axes. By employing a single‐step Raman measurement (rather than armchair or zigzag), we probe two Raman modes simultaneously to quantify the material's thermal anisotropy. The anisotropy in ReS_2_ is distinct from that in other well‐known anisotropic materials such as SnSe_2_, PdTe_2_, and BP, which typically exhibit uniaxial or biaxial in‐plane anisotropy. ReS_2_ (ReSe_2_) features a non‐orthogonal basis leading to wandering or tilting of optical axes within the plane. In this work, we perform a systematic vibrational analysis to elucidate the layer‐dependent anisotropic behavior due to the wandering of the characteristic vibrational axes in ReS_2_ crystals.

In this work, we investigated in‐plane anisotropic thermal transport (ATT) in the triclinic *1T′* layered ReS_2_ system. We experimentally probed the ATT through Raman thermometry [34, 35, 36], and also validated the observation with first‐principles calculations and symmetry analysis. Prior to the determination of in‐plane ATC and ATEC in few‐layer ReS_2_, we performed angle‐resolved polarized Raman (ARPR) Spectroscopy to identify the in‐plane crystallographic orientations. Then, Raman thermometry (power‐ and temperature‐dependent) was employed to extract the in‐plane ATC and ATEC in suspended ReS_2_ flakes, having thicknesses of *5–8L*. This study investigates the in‐plane ATC and ATEC in few‐layer ReS_2_ using combined experimental and theoretical approaches, and systematically analyzes the thickness dependence of anisotropy. To the best of our knowledge, this study also provides the first experimental demonstration of anisotropic TECs in an anisotropic 2D system. We believe that this study opens up a new paradigm in anisotropic 2D systems for further optoelectronic and thermoelectric device applications.

## 2 Results and Discussion

### 2.1 In‐Plane Anisotropy

**FIGURE 1:**
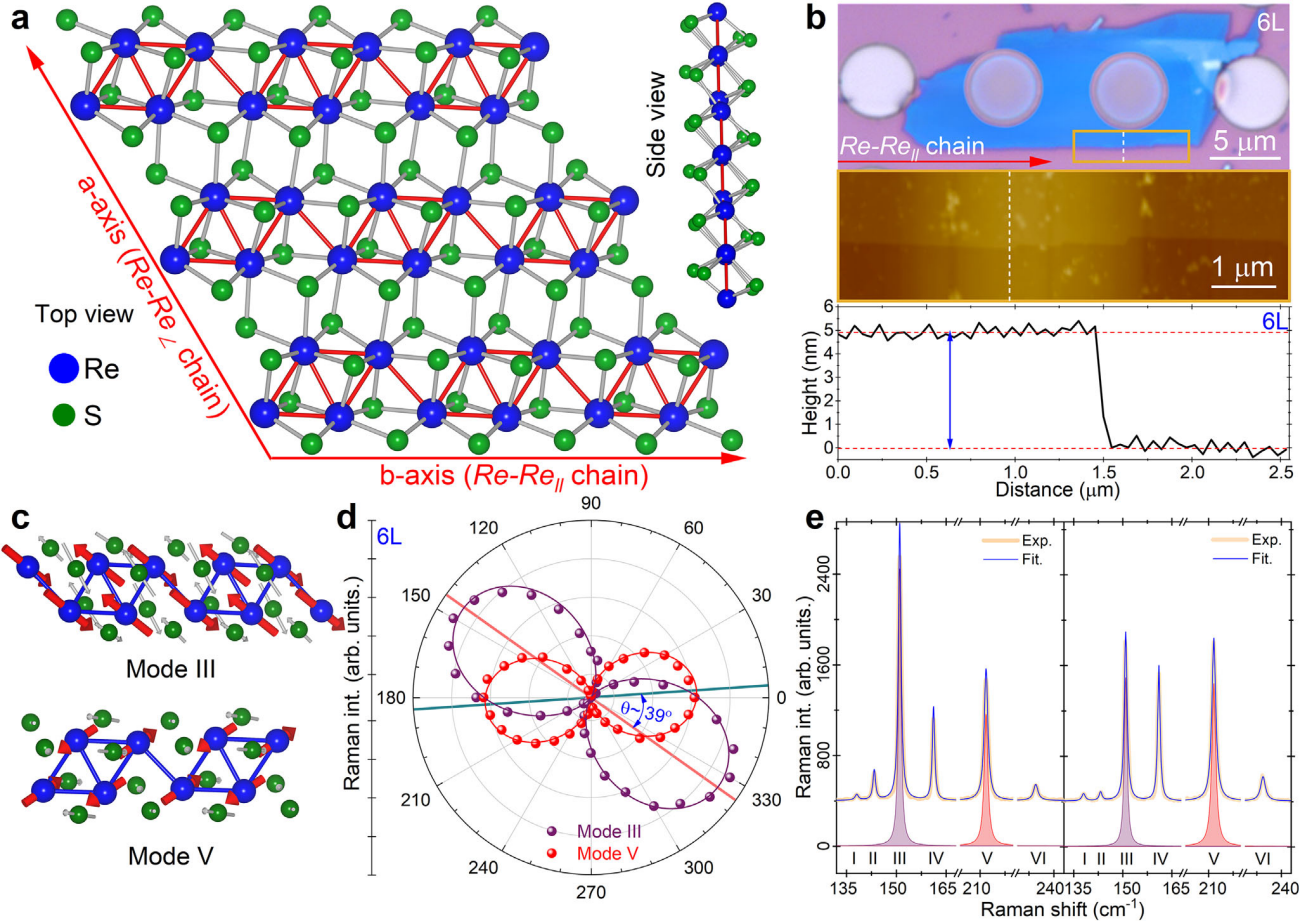
(a) Crystal structure of *1L*‐ReS_2_ (top and side view). The crystallographic ‘*b*’‐axis and ‘*a*’‐axis are parallel to the *Re‐Re* diamond chain (red) (*Re* − *Re*_∥_) and at a certain angle with the *Re‐Re* chain(*Re* − *Re*_∠_), respectively. (b) OM image (upper panel) of a suspended *6L*‐ReS_2_ flake positioned over an array of holes. The corresponding AFM topography (middle panel) and the height profile (bottom panel). (c) Vibrational eigenvectors correspond to the Raman modes III and V. (d) ARPR intensities of modes III (violet sphere) and V (red sphere) of *6L*‐ReS_2_, exhibiting an angular deviation of 39° between their maximum intensity distribution. (e) Polarized Raman spectra of *6L*‐ReS_2_, taken at 330° and 0° polarizer angles, respectively. Modes are fitted with a multi‐Lorentzian (solid line). Modes III (violet) and V(pink) are highlighted with solid fill.

Unlike group VI TMDCs, the group VII compound like ReX_2_ (X = S, Se) possesses additional electrons that promote the formation of Re‐Re bonds, driving a *Peierls* distortion [22, 37, 38]. This distortion transforms the ideal trigonal (*1T*) lattice into a distorted *1T′* configuration, lowering the symmetry to a triclinic structure having a space group $P\bar{1}$, in which the principal crystallographic axes are non‐orthogonal. Figure 1a illustrates the crystal structure of monolayer (*1L*) ReS_2_, consisting of a Re‐layer sandwiched between two S‐layers, belonging to the *C*_i_ point group preserving inversion symmetry [39, 40]. The *Re*‐*Re* diamond chain direction is usually defined as the ‘*b*’‐axis (*Re* − *Re*_∥_), and the other in‐plane principal axis is designated as the ‘*a*’‐axis (*Re* − *Re*_∠_), having an angle of either 60° (or 120°) with the ‘*b*’‐axis. To investigate the in‐plane anisotropic thermal conductivity, as well as the thermal expansion coefficient of few‐layer suspended ReS_2_, the flakes were transferred onto a patterned (array of holes with a diameter ∼ 5 µm) SiO_2_(290 nm)/Si substrate by the dry transfer method (see the methods section). Figure 1b (upper panel) illustrates the optical microscopy (OM) image of a suspended ReS_2_ flake (*6L*). Topography and height profile (confirmed by atomic force microscopy (AFM)) are shown in the middle and lower panels of Figure 1b. For other thicknesses (*5L*, *7L*, and *8L*), the OM images, AFM topography, and corresponding height profiles are shown in Figure S1a–i. According to the group‐theoretical symmetry analysis, the 36 Γ‐point phonon modes decompose into the irreducible representations Γ = 18(*A_g_* + *A_u_​*), where the *A_g_* modes are Raman active. The 18 Raman‐active *A_g_* modes of ReS_2_ are observed in the frequency range of 125–450 cm^−1^. The Raman spectra of ReS_2_ flakes (*5*–*8L*) are shown in Figure S2a–d. For simplicity, the observed peaks are sequentially labeled as modes I‐XVIII. It should be noted that ReS_2_ has two different polymorphs, named *AA* and *AB* [41, 42]. In our measurement, we have chosen the *AA* phase, which was confirmed through the peak separation (∼13 cm^−1^) of modes I and III (see Table S1) [42, 43]. Among the 18 Raman‐active modes, we primarily focused on mode III (∼151 cm^−1^) and mode V (∼212 cm^−1^), which capture the essence of *Re‐Re* atomic chain vibrations (*Re* − *Re*_∠ _and *Re* − *Re*_∥_) along the crystallographic ‘*a*’ and ‘*b*’‐axes, respectively, as illustrated by the corresponding vibrational eigenvectors in Figure 1c. The resultant atomic displacements of modes III and V are predominantly aligned with these major crystal axes, making them highly sensitive to in‐plane anisotropy.

Prior to the measurement of ATC, we performed ARPR for a few layers of suspended ReS_2_ for the confirmation of crystallographic axes along with the in‐plane anisotropy. Due to the reduced symmetry, all Raman modes exhibit distinct polarization dependencies. Among them, the relative directions of the maximum‐intensity polarization of the Raman modes III (∼151 cm^−1^) and V (∼212 cm^−1^) can be used to determine the ‘*a*’ and ‘*b*’‐axes directions. Figure 1d shows the polar intensity distribution of modes III and V (for *6L*‐ReS_2_) in the ARPR measurement, which shows an angular separation of ∼39° between the two modes.

For other thicknesses, this angular separation varies with layer number, being ∼36°, 42°, and 45° for *5L*, *7L*, and *8L*, respectively, as shown in Figure S3a–c. The intensity distribution was fitted with Raman tensor analysis (Supporting Information Note I). The maximum intensity variation of these two modes (for mode III at 330° and mode V at 0°) for the specific case of *6L*‐ReS_2_ is shown separately in Figure 1e. The polarization maxima of Raman modes III and V for different polarizer orientations across *5–8L* samples are summarized in Figure S4. Although the crystallographic ‘*a*’ and ‘*b*’‐axes of ReS_2_ are separated by 60°, the intensity maximum angular separation of modes III and V is less than 60°. Similar observations have also been reported in earlier studies [24, 44, 45]. This could be due to the mismatch between the optical axis and crystallographic axis, as explained by Novosolve et al. [45]. Our observations also align with the previous results. Therefore, the orientation of mode III and mode V maxima will represent the optical anisotropy in the in‐plane orientation of a specific thickness of ReS_2_. As a result, by monitoring the frequency shifts of phonon modes III and V as a function of laser power and temperature, the in‐plane anisotropic thermal conductivity of few‐layer ReS_2_ can be determined along the ‘*a*’ and ‘*b*’‐axes, respectively.

### 2.2 In‐Plane Anisotropic Thermal Conductivity

#### 2.2.1 Experimental Results

One of the most widely employed non‐contact techniques to investigate the thermal properties of 2D materials is Raman thermometry, where a focused laser beam serves as a localized heat source, which increases the local temperature of the sample, and the resulting temperature rise is monitored through Raman spectroscopy. The Raman‐based thermometry relies on the inelastic scattering of the excitation laser, where the frequency shift of a temperature‐calibrated Raman mode is employed as a sensitive probe of the local temperature in the suspended sample. Therefore, the power‐ and temperature‐dependent Raman studies have been carried out to determine the thermal conductivity. Herein, Raman thermometry was performed using a 632.8 nm continuous‐wave (CW) laser as the excitation source. The laser was focused to a spot size of r_0_ ≈ 0.76 µm (see spot size calculation [46], Figure S5) at the center of suspended ReS_2_ flakes with thicknesses ranging from *5–8L*. The fraction of incident laser power absorbed by the 2D material plays a decisive role in the effective heating and thereby influences the thermal conductivity values determined from the optothermal Raman technique. For anisotropic materials, the absorption of incident laser power along the crystallographic axis (‘*a*’ or ‘*b*’‐axis) differs due to a change in the refractive index along the principal optical axis [45]. In few‐layer ReS_2_, anisotropic absorption behavior is evident across the examined energy range, stemming from the combined effects of refractive index anisotropy and thickness‐dependent optical absorption (see Figure S6 and Table S2). To further validate the anisotropic absorption behavior, heat propagation simulations using the finite element method were performed (see Figure S7 and Note II). Therefore, to estimate the in‐plane ATC in ReS_2_, we have analyzed modes III and V with the variation of absorbed power and temperature, which leads to the anisotropic heat propagation along the two in‐plane crystallographic axes ‘*a*’ and ‘*b*’.

**FIGURE 2:**
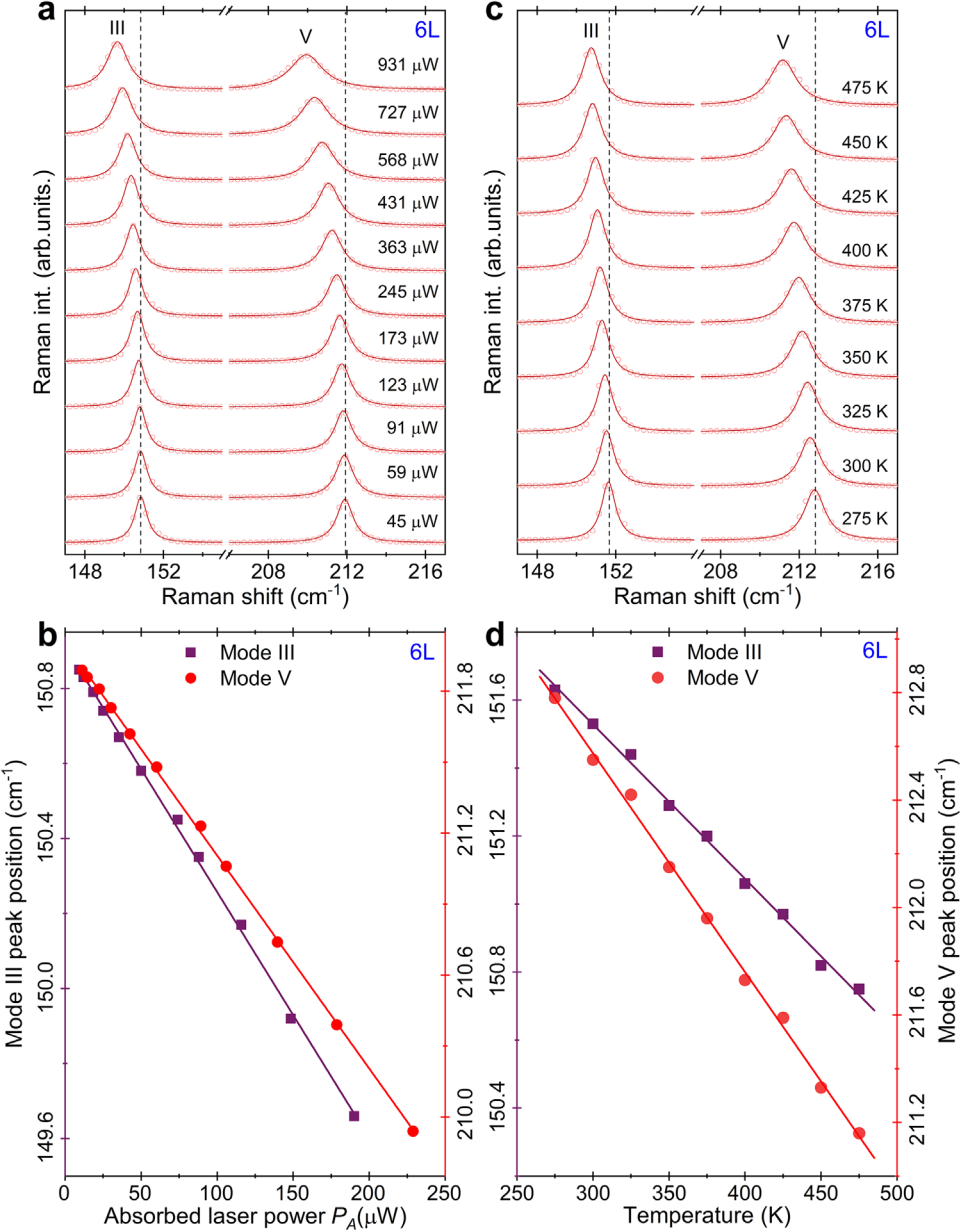
(a) Raman spectra of a *6L*‐ReS_2_ flake acquired under varying incident laser powers ranging from 45 to 931 µW, highlighting the frequency shifts of modes III and V. The black dotted lines indicate the reference peak positions of modes III and V with reference to 45 µW. (b) Absorbed laser power‐dependent evolution of the peak positions of Raman modes III (red circles) and V (violet rectangles). (c) Raman spectra of the *6L*‐ReS_2_ flake measured over the temperature range of 275–475 K. The black dotted lines indicate the reference peak positions of modes III and V with reference to 275 K. (d) Temperature‐dependent evolution of the peak positions of Raman modes III (red circles) and V (violet rectangles). The Solid lines represent the linear fit of the experimental data.

**FIGURE 3:**
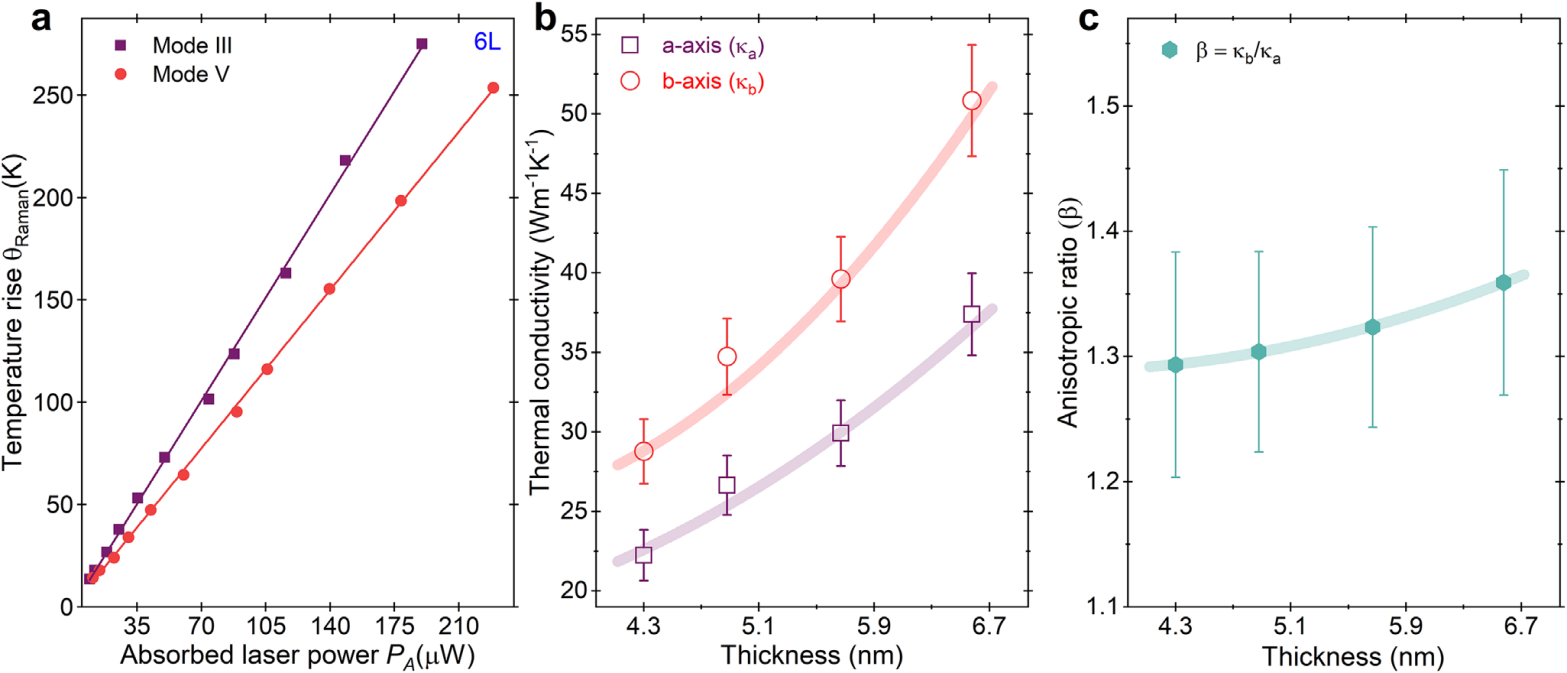
Thermal conductivity measurement of a few layers of ReS_2_ by Raman thermometry. (a) Absorbed laser power (P_A_) dependent temperature rise (θ_Raman_) of the *6L*‐ReS_2_ flake along ‘*a*’ and ‘*b*’‐axes. The solid lines represent linear fits to the experimental data. (b) Extracted in‐plane anisotropic thermal conductivities of *5L*–*8L* of ReS_2_ along ‘*a*’ and ‘*b*’‐axes, respectively, as a function of thickness. (c) Anisotropic ratio (β = κ_*b*_/κ_*a*_)of the in‐plane thermal conductivity for different thicknesses of ReS_2_ flakes. The solid lines in (b) and (c) represent the data points with error bars and using a guide‐to‐the‐eye.

In Figure 2a, the peak frequencies of modes III and V in the *6L*‐ReS_2_ (suspended) flake are shown as a function of incident laser power. Similarly, the results on flakes with different layer numbers (*5L*, *7L*, and *8L*) are shown in Figure S8. An increase in laser power corresponds to a higher local temperature rise (ΔT = θ_Raman_), leading to a red shift of both modes. Notably, the first‐order power coefficient for mode V (− 0.9 × 10^−2^ cm^−1^ µW^−1^) exhibits a larger red shift compared to mode III (− 0.655 × 10^−2^ cm^−1^µW^−1^), indicating its stronger temperature sensitivity as a function of absorbed laser power, P_A_, as depicted in Figure 2b (for other thicknesses, see Figure S9 and Table S3). In previous studies, the in‐plane ATC of BP [33] and Td‐WTe_2_ [32] was determined by analyzing a single Raman mode (∼ 464 cm^−1^ for BP and ∼212 cm^−1^ for Td‐WTe_2_). In these approaches, the sample was either aligned along the zigzag or armchair crystallographic axes, or the laser polarization was oriented along these axes, to extract the ATC. In contrast, our approach for ReS_2_ offers a distinct advantage: neither sample alignment along crystallographic axes nor laser polarization matching is required. This is because Raman modes III and V intrinsically correspond to vibrations along the in‐plane crystallographic ‘*a*’ and ‘*b*’ axes. Thus, by independently analyzing the power‐ and temperature‐dependent phonon shifts of these two modes in a single measurement, the ATC along both axes can be directly obtained. For validation of our method against the earlier approach on Td‐WTe_2_, we performed Raman measurements (for *8L*) with the laser polarization aligned along the ‘*a*’ and ‘*b*’ axes and compared the results with those obtained using unpolarized excitation for modes III and V under power‐dependent conditions. Both results showed excellent agreement (see Figure S10). Hence, we conclude that our method offers a more convenient and straightforward way to extract the ATC in ReS_2_. Temperature‐dependent Raman measurements were conducted on *6L*‐ReS_2_ to investigate the phonon frequency shifts of modes III and V in the temperature range 275–475 K, as shown in Figure 2c (for other thicknesses, see Figure S11). To minimize laser‐induced heating effects, the laser power was kept to a minimum during the temperature‐dependent study. The individual Raman spectra (in Figure 2a,c) were fitted using a multi‐Lorentzian function to accurately determine the peak positions. As the temperature increases, the phonon interacts more due to the anharmonic term in the lattice potential, which causes a softening of optical phonons that leads to a red shift in phonon modes [47]. For *6L*‐ReS_2_, the first‐order temperature coefficient of Mode V(− 0.815 × 10^−2^ cm^−1^K^−1^) exhibits a larger red‐shift than Mode III(− 0.455 × 10^−2^ cm^−1^K^−1^), which is shown in Figure 2d (for other thicknesses, see Figure S12 and Table S3). This mismatch eventually leads to the in‐plane ATC along the optic axes. Mode V is associated with weaker and more anisotropic bonds, yields a higher value of the Grüneisen parameter, and stronger acoustic phonon coupling. Therefore, it enhances the anharmonic phonon‐phonon interactions that drive a greater temperature‐dependent frequency softening for mode V compared to mode III. Using these temperature coefficients, the laser power‐induced red‐shift of the Raman modes III and V is translated into the corresponding local temperature rise (θ_Raman_) as a function of the absorber laser power (P_A_). In Figure 3a, the local temperature rise along modes III and V shows distinct anisotropy with varying absorbed power (for other thicknesses, see Figure S13).

To extract the in‐plane ATC along both axes, linear fits of modes III and V were applied to the extracted ΔT as a function of absorbed laser power (*P_A_*), yielding the slope $\left(\frac{\partial T}{\partial P_{A}}\right)$, which was subsequently used in the following expression [48],

(1) $$\kappa_{a,b}=\alpha \left(\frac{1}{2\pi t}\right){\left(\frac{\partial T}{\partial P_{A}}\right)}_{a,b}^{-1}\ln\left(\frac{R}{r_{0}}\right)$$

where *t* represents the thickness of the ReS_2_ flake, *R* and *r*_0_ are the radii of the hole and laser spot, respectively, and α is a dimensionless prefactor expressed as a function of *R*/*r*_0_. Under our experimental conditions, α ∼ 1 [48]. Using Equation (1), the in‐plane thermal conductivities along the ‘*a*’ and ‘*b*’‐axes were extracted for ReS_2_ flakes with thicknesses ranging from *5L*–*8L*, as presented in Figure 3b. With increasing thickness, the thermal conductivity shows an upward trend (along ‘a’ and ‘b’‐axes $\kappa_{a}^{5L}$ = 22.25 ± 1.59 Wm^−1^K^−1^ to $\kappa_{a}^{8L}$ = 37.4 ± 2.59 Wm^−1^K^−1^ and $\kappa_{b}^{5L}$ = 28.78 ± 2.03 Wm^−1^K^−1^ to $\kappa_{b}^{8L}$ = 50.83 ± 3.50 Wm^−1^K^−1^), primarily due to the suppression of phonon‐boundary scattering. The total relative error in *κ* was derived by propagating statistical and systematic experimental errors (see Note III). Furthermore, the anisotropy ratio (β) also increases with thickness (β^5*L*^ ∼ 1.29 to β^8*L*^ ∼ 1.36), indicating that the influence of intrinsic phonon anisotropy progressively outweighs that of extrinsic scattering, as illustrated in Figure 3c. The in‐plane ATC along ‘*b*’ and ‘*a*’‐axes and their anisotropic ratio (β) for different thicknesses have been tabulated in Table S4.

#### 2.2.2 Theoretical Aspect

**FIGURE 4:**
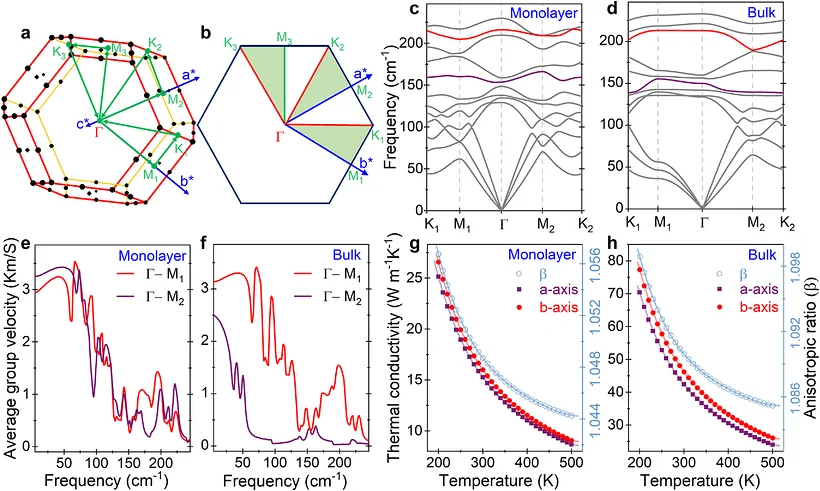
Theoretical calculation of thermal anisotropy in monolayer and bulk ReS_2_. (a‐b) High‐symmetry paths, Γ–M_1_ (b^*^‐axis) and Γ–M_2_ (a^*^‐axis), in the Brillouin zones (BZ) of the bulk and monolayer systems. (c‐d) Harmonic phonon dispersion spectra, with the violet and red highlighted dispersions corresponding to modes III and V, respectively. (e‐f) Mode‐dependent average phonon group velocities along the considered crystallographic directions. (g‐h) Thermal conductivity as a function of temperature along the ‘*a*’(violet rectangles) and ‘*b*’ (red circles) axes for monolayer and bulk ReS_2_. The open blue spheres represent the anisotropy ratio (β) of the in‐plane thermal conductivity for both systems.

The strong in‐plane anisotropy arising from the distorted *1T'* crystal structure directly influences the phonon dispersion and, consequently, the thermal conductivity of ReS_2_. To quantitatively assess the degree of thermal anisotropy in layered ReS_2_, we first examine its phonon behavior in both the monolayer and bulk limits. The high‐symmetry paths of interest constructed within the Brillouin zones (BZ) of these systems, along the in‐plane reciprocal axes (Γ‐M_1_ and Γ‐M_2_ directions), are illustrated in Figure 4a,b. The phonon spectra along these two directions, presented in Figure 4c,d, respectively, exhibit distinctly different slopes and reveal directional inhomogeneity in both systems that extends across the full frequency range. The low‐energy optical modes (< 240 cm^−1^) are mostly dominated by the vibrations due to Re atoms, as seen by the phonon density of states in Figure S14. Particularly, the dispersion of modes III and V, associated with the axial vibrations, primarily dominated by asymmetrically buckled *Re‐Re* chains along the ‘*a*’ and ‘*b*’‐axes, are highlighted in Figure 4c,d. As referred in the previous section, the distorted trigonal symmetry of the ReS_2_ crystal provides a coupled vibrational basis [49], where the real‐space vibrations along the *Re*‐*Re* chain (*Re* − *Re*_∥_) and those at a certain angle to the *Re*‐*Re* chain (*Re* − *Re*_∠_) can be associated with the phonon behavior along the reciprocal directions Γ‐M_2_ and Γ‐M_1_, respectively. To verify this correspondence, we calculated the angular separation of the vibrational eigenvectors for modes III and V, which was measured to be ∼ 36° for the monolayer and ∼ 42° for the bulk. These values are in excellent agreement with the experimental results obtained in this work. The phonon group velocity (*v_g_*), extracted from the slopes of the dispersion curves, directly reflects the lattice anisotropy. The frequency‐dependent average *v_g_* captures the cumulative anisotropy in the reciprocal space, which is written as [50, 51],

(2) $$v_{av}\left(\omega \right)=\frac{v_{i}^{2}}{\left|v\right|}\left(\omega \right)={\left(\frac{\Delta q}{2\pi }\right)}^{3}\sum_{\lambda ,i}\frac{\frac{{\left(v_{i}^{\lambda }\right)}^{2}}{\left|v_{i}^{\lambda }\right|}\delta \left(\omega -\omega_{\lambda }\right)}{\sum_{\lambda }g_{\lambda }\left(\omega \right)}$$

where, $v_{i}^{\lambda }$ is the *i*‐th component of the group velocity of phonon mode λ at a wavevector ***q*** and *g*_λ_denotes the phonon density of states. There is an overall contrast in *v_av_* coming from vibrations along*Re* − *Re*_∥_ and*Re* − *Re*_∠_, mostly in the low‐frequency region (< 240 cm^−1^), with a larger directional difference in the bulk crystal than the monolayer, as seen in Figure 4e,f, respectively. For comparison, the experimentally calibrated modes III and V yield *v_av_* values of ∼ 427 (434) m/s, ∼ 295 (436) m/s for monolayer, and ∼ 325 (472), ∼ 212 (473) m/s for bulk along Γ‐M_2_ (Γ‐M_1_). This thickness‐dependent behavior is further examined by considering *AA‐*stacked ReS_2_ systems up to *5L*, as shown in Figure S15. Since our study focuses only on the in‐plane anisotropy, while additional layers introduced along the crystallographic c direction introduce very limited change in *v_av_* along the two considered in‐plane directions. However, the difference in the absolute values of *v_av_* between Γ‐M_2_ and Γ‐M_1_, suggests that vibrational anisotropy is more pronounced once we move toward the bulk limit. The difference in absolute values of *v_av_* suggests that vibrational anisotropy is more pronounced if we move toward the bulk limit. Within the range of ∼ 240 cm^−1^, higher *v_av_* along Γ‐M_1_ suggests that phonons in this direction of *Re* − *Re*_∠_ experience less scattering and therefore make stronger contributions to the cumulative thermal conductivity. However, above this threshold (> 240 cm^−1^), there is a drop in *v_av_*, primarily due to strong scattering from lone‐pair electron repulsion of the lighter S atoms [52]. The TC calculated from phonon‐scattering theory (Figure 4g,h) closely follows the trends observed in *v_av_*. At room temperature (300 K), the thermal conductivity values are approximately 15.3 (16.4) W m^−^^1^ K^−^^1^ for the monolayer and about 42.4 (46.2) W m^−^^1^ K^−^^1^ for the bulk, along Γ‐M_2_ (Γ‐M_1_), respectively.

The anisotropy ratio (β), calculated as $TC_{\Gamma -M_{1}}:TC_{\Gamma -M_{2}}$, is ∼1.05 for the monolayer, increasing up to ∼1.1 for the bulk. This indicates a higher degree of directionality of the planar lattice dynamics in the bulk case. These results are consistent with experimental observations revealing that TC in both systems is highly anisotropic, which strengthens with increasing layer number and temperature.

### 2.3 Anisotropic Thermal Expansion Coefficient

The thermal expansion coefficient (TEC) in a 2D system can be described through the quasi‐harmonic approximation (QHA). Within this framework, if all vibrational modes are initially treated as harmonic, the free energy of the system can be expressed analytically. The TEC can be derived as follows [53, 54],

(3) $$\alpha =\frac{1}{l_{0}^{2}{\left.\frac{\partial^{2}E}{\partial l^{2}}\right|}_{0}}\sum_{k,m}\gamma_{k,m}\;c_{v}\left(k,m\right)$$

**FIGURE 5:**
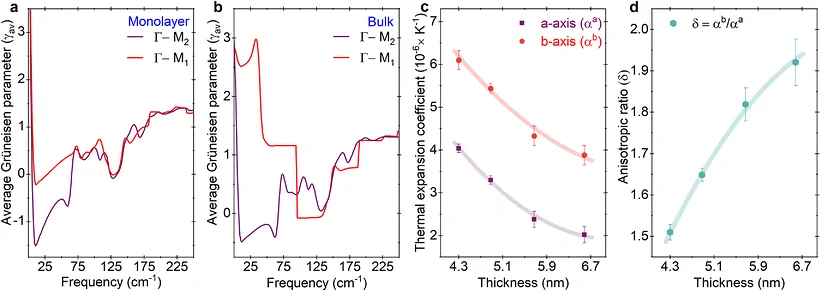
(a‐b) Average Grüneisen parameter of *1L* and bulk ReS_2_ along Γ‐M_1_ path and Γ‐M_2_ path, respectively. The violet line and red line show the Γ‐M_1_ and Γ‐M_2_ paths over the frequency range 5–245 cm^−1^. (c) Extracted in‐plane ATECs of 5–8L of ReS_2_ along ‘*a*’ and ‘*b*’‐axes, respectively. (d) Anisotropic ratio (δ = α^*b*^/α^*a*^)of the in‐plane TEC along ‘*b*’ and ‘*a*’‐axes as a function of ReS_2_ flake thickness. The solid lines in (c) and (d) represent the data points with error bars and using a guide‐to‐the‐eye.

Here, the subscript ‘0’ denotes the ground state, *l* represents the lattice constant, and *E* is the total energy of the system. The term *c_v_* (**k**, m) corresponds to the contribution to the heat capacity from the *m^th^* phonon branch at wavevector **k**. The quantity γ_*k*,*m*_ is the Grüneisen parameter, which quantifies the degree of phonon anharmonicity, defined as [53],

(4) $$\gamma_{k,m}=\frac{-l_{0}}{\omega_{0,k,m}}{\left.\frac{\partial \omega_{k,m}}{\partial l}\right|}_{0}$$

where, ω_***k***,*m*_​ denotes the phonon frequency of the *m^th^* branch at wavevector **k**, while ω_0, ***k***, *m*_ ​ represents the corresponding frequency in the ground state configuration. Theoretical estimation of the average Grüneisen parameter,γ_*av*_(ω_*m*_)was obtained by performing a frequency‐dependent density regression over the two high‐symmetry directions of the BZ as,

(5) $$\gamma_{av}\left(\omega_{m}\right)=\frac{\sum_{k}\gamma_{k,m}\delta \left(\omega_{m}-\omega_{k,m}\right)}{g\left(\omega_{m}\right)}$$

where *g*(ω_*m*_) is the phonon density of states. As shown in Figure 5a,b, the low‐frequency optical phonon modes exhibit strong temperature‐dependent volume responses in the quasi‐anharmonic regime, which gradually saturate in the higher‐frequency regime. As already established through phonon dispersion, the vibrations along the closely spaced diamond‐shaped *Re* − *Re*_∥_ and *Re* − *Re*_∠_ are the origin of thermal anisotropy in layered ReS_2_. These dynamically distinct vibrations give rise to inhomogeneous electron localization along the ‘*a*’ and ‘*b*’‐axes, which increases with layer thickness. This leads to the enhancement of γ_*av*_(ω_*m*_)along the Γ‐M_2_ than the Γ‐M_1,_ which replicates the behavior of TEC along the crystallographic ‘*b*’ and ‘*a*’‐axes, respectively. In addition, the mode‐specific Grüneisen parameters (γ_*k*,*m*_) for modes III and V along the selected BZ path exhibit distinctly different slopes between the monolayer and bulk ReS_2_, as highlighted in Figure S16.

To elucidate the influence of anisotropic TEC on the phonon frequency shifts, we carried out a detailed symmetry and perturbation analysis of few‐layer ReS_2_. Based on the basis functions of the *C_i_* point group, the deformation potential was constructed by using the corresponding character table (see Table S5 and Supporting Information Note IV). Owing to the inherent anisotropy of ReS_2_, the stress‐ and temperature‐dependent phonon frequency shifts along the ‘*a*’ and ‘*b*’‐axes can be expressed as,

(6) $$\Delta \omega_{a,b}^{A_{g}}=K^{a,b}\sigma_{a,b}+A^{a,b}\Delta T$$

where *K^b^* and *K^a^* are the stress coefficients along ‘*b*’ and ‘*a*’‐axes, respectively. The stress coefficients along both axes (*K* ^*b*^ ∼ ‐0.56 cm^−1^GPa^−1^ and *K* ^*a*^ ∼ ‐0.428 cm^−1^GPa^−1^) for few‐layer ReS_2_ were calculated by using the gauge factor values from the report [55]. *A^b^* and *A^a^* are the temperature coefficients along ‘*b*’ and ‘*a*’‐axes, for the suspended region, respectively. σ_*a*,*b*_ is the in‐plane (along either ‘*a*’ or ‘*b*’‐axis) thermal stress within the ReS_2_ flakes. The hole has a diameter (D) of ∼ 5 µm. Since the suspended few‐layer ReS_2_ possesses a very high aspect ratio (D/t ∼756‐1154), any compressive stress present can be relaxed through buckling. Therefore, the suspended region is expected to remain nearly stress‐free (for details, see Supporting Information Notes V, VI and Figure S17). The strain‐stress relations of the flakes supported by SiO_2_(290 nm)/Si substrate can be expressed as,

(7) $$\varepsilon_{SiO_{2}}^{a,b}=\alpha^{a,b}\Delta T+\frac{1-\nu }{E_{a,b}}\sigma_{a,b}=\alpha_{SiO_{2}}\Delta T$$

where, $\varepsilon_{SiO_{2}}^{a,b}$ is the in‐plane (along either ‘*a*’ or ‘*b*’‐axis) thermal strain within the ReS_2_ flakes on the SiO_2_(290 nm)/Si substrate. *α* ^*a*,*b*^and $\alpha_{SiO_{2}}$ are the in‐plane TEC of ReS_2_ flakes (along either ‘*a*’ or ‘*b*’‐axis) and SiO_2_, respectively and *v* represents the Poisson ratio of ReS_2_. *E_a_* and *E_b_* are the Young's moduli along the ‘*a*’ and ‘*b*’‐axes. The anisotropic Young's modulus of few‐layer ReS_2_, corresponding to both the principal in‐plane axes (*E_a_* ∼ 201 GPa and *E_b_* ∼ 127 GPa), was adopted from the earlier report [56]. So, the in‐plane (along either ‘*a*’ or ‘*b*’‐axis) thermal stress can be expressed as,

(8) $$\sigma_{a,b}=\frac{E_{a,b}\left(\alpha_{SiO_{2}}-\alpha^{a,b}\right)}{1-\nu }\Delta T$$

By substituting the Equation (8) into the Equation (6), we obtained the temperature‐dependent frequency shift along ‘*a*’ or ‘*b*’‐axis as,

(9) $$\Delta \omega_{SiO_{2}}^{a,b}=K^{a,b}\frac{\left(\alpha_{SiO_{2}}-\alpha^{a,b}\right)E_{a,b}}{1-\nu }\Delta T+A^{a,b}\Delta T$$

where, $\Delta \omega_{SiO_{2}}^{a}\;\mathrm{and}\;\;\Delta \omega_{SiO_{2}}^{b}$ are the temperature‐dependent frequency shifts of substrate (SiO_2_/Si) supported ReS_2_ flakes along ‘*a*’ and ‘*b*’‐axes, respectively. The temperature coefficients $A_{SiO_{2}}^{a},A_{SiO_{2}}^{b},A^{a},\mathrm{and}\;A^{b}$ can be measured from the temperature‐dependent Raman experiments (see Figures S18, S19, and Table S6). Therefore, the TEC along ‘*a*’ or ‘*b*’‐axis can be expressed as a function of temperature coefficients,

(10) $$\alpha^{a,b}=\alpha_{SiO_{2}}-\frac{1-\nu }{E_{a,b}K^{a,b}}\left(A_{SiO_{2}}^{a,b}-A^{a,b}\right)$$

Using Equation (10), the in‐plane TEC along ‘*a*’ and ‘*b*’‐axes were extracted for ReS_2_ flakes with thicknesses ranging from *5–8L*, as presented in Figure 5c. With increasing thickness, the TEC shows a decreasing trend (along ‘*a*’ and ‘*b*’‐axes $\alpha_{a}^{5L}$ = (4.04 ± 0.09)×10^−6^ K^−1^ to $\alpha_{a}^{8L}$ = (2.02 ± 0.18)×10^−6^ K^−1^ and $\alpha_{b}^{5L}$ = (6.1 ± 0.22)×10^−6^ K^−1^ to $\alpha_{b}^{8L}$ = (3.88 ± 0.22)×10^−6^ K^−1^), primarily due to the absence of interlayer vdW interaction [57, 58]. Furthermore, the anisotropy ratio(δ) also increases with thickness (δ^5*L*^ ∼ 1.51 to δ^8*L*^ ∼ 1.92), as illustrated in Figure 5d. The in‐plane anisotropic TEC along ‘*b*’ and ‘*a*’‐axes and their anisotropic ratio(δ)for different thicknesses are summarized in Table S7. In summary, the ATEC in few‐layer ReS_2_ originates from the inherent in‐plane structural anisotropy associated with the directional Re‐Re chain, resulting in distinct thermal expansion along *Re* − *Re*_∥_ and *Re* − *Re*_∠_.

## 3 Conclusion

In summary, we probed the in‐plane ATC and ATEC of suspended few‐layer ReS_2_ flakes using Raman spectroscopy and analyzed the thickness‐dependent variation of the anisotropic ratio. This is the first experimental demonstration of ATEC in 2D layered materials. Following the confirmation of the crystallographic axis of ReS_2_ through ARPR measurements, thermal conductivity (along ‘*a*’ and ‘*b*’‐axis) was extracted from Raman thermometry data. The extracted thermal conductivity of ReS_2_ along ‘*a*’‐axis is $\kappa_{a}^{5L}$ = 22.25 ± 0.29 Wm^−1^K^−1^ to $\kappa_{a}^{8L}$ = 37.4 ± 0.32 Wm^−1^K^−1^ for 5–8L thicknesses. Whereas, along ‘*b*’‐axis the values varies from $\kappa_{b}^{5L}$ = 28.78 ± 0.23 Wm^−1^K^−1^ to $\kappa_{b}^{8L}$ = 50.83 ± 0.38 Wm^−1^K^−1^ corresponding to *5–8L*. Depending on the thickness, this yields an ATC ratio (β) in the range of ∼1.29 to 1.36. The microscopic origin of in‐plane ATC can be attributed to the phonon dispersion characteristics, group velocities, and scattering rates associated with lattice vibrations along *Re* − *Re*_∥_ and *Re* − *Re*_∠_, as obtained from first‐principles calculations. Also, we have estimated ATECs along two different axes (along ‘*a*’ and ‘*b*’‐axes $\alpha_{a}^{5L}$ = (4.04 ± 0.09) × 10^−6^ K^−1^ to $\alpha_{a}^{8L}$ = (2.02 ± 0.18) × 10^−6^ K^−1^ and $\alpha_{b}^{5L}$ = (6.1 ± 0.22) × 10^−6^ K^−1^ to $\alpha_{b}^{8L}$ = (3.88 ± 0.22) × 10^−6^ K^−1^) for different layer thicknesses (*5–8L*). Based on symmetry analysis, we demonstrated that the TEC influences the Γ‐point optical phonon frequency through the combined effects of in‐plane thermal stress and anisotropic temperature coefficients, originating from thermal mismatch and free expansion. The estimated ATEC ratio (*δ*) ranges from ∼1.52 to 1.91, depending on the thickness. The calculated average Grüneisen parameter elucidates this ATEC by quantifying the thermal response to volume changes from the monolayer to the bulk limit. This finding provides a foundation for constructing anisotropic optoelectronic devices in other low‐symmetry 2D systems.

## 4 Experimental Section

### 4.1 Sample Fabrication

High‐quality single‐crystal ReS_2_ (commercially obtained from 2D Semiconductors) was used for all measurements. Bulk ReS_2_ crystals were mechanically exfoliated onto a SiO_2_(290 nm)/Si substrate using the standard scotch tape method. In order to achieve suspended few‐layer ReS_2_ flakes, circular holes with a diameter of ∼ 5 µm were patterned on the SiO_2_(290 nm)/Si substrate by electron beam lithography (EBL) followed by reactive ion etching (RIE). The exfoliated ReS_2_ flakes were then transferred onto the array of holes using a standard dry transfer method.

### 4.2 Optical Microscopy (OM) and Atomic Force Microscopy (AFM)

Bright‐field OM imaging was carried out using a Nikon ECLIPSE LV100ND microscope, equipped with a 100× objective lens (NA = 0.95). During image acquisition, the exposure time was fixed at 100 ms, and the white balance was calibrated using a predefined R: G: B ratio. The surface morphology of few‐layer ReS_2_ was further examined by AFM (Park Systems NX10) employing an Al‐coated cantilever (PPP‐NCHR). All the AFM measurements were performed in non‐contact mode with a scan rate of 0.3 Hz.

### 4.3 Raman Thermometry

Temperature‐ and power‐dependent Raman spectra were acquired using a confocal Raman spectrometer (LabRam 800, Horiba Jobin Yvon) operated in backscattering geometry with a 50× long working distance objective (NA = 0.5). A 632.8 nm laser was employed as the excitation source, yielding a spot size of ∼1.53 µm on the sample surface (see Figure S5, Supporting Information, for spot‐size determination). Spectra were collected using a 1800 lines/mm grating. For temperature‐dependent measurements, the samples were mounted in a liquid‐nitrogen‐cooled vacuum cryostat (Linkam). The flakes were affixed to a thin glass plate using silver paste to ensure efficient thermal coupling with the stage, and a stabilization time of 10 min was maintained at each set temperature. Raman measurements were performed on both supported and suspended regions of the flakes, yielding consistent results (see Supporting Information). The temperature rise was quantified as $\Delta T\left(\theta_{Raman}\right)=\left(\frac{\upsilon_{P_{A}}-\upsilon_{P_{A}=0})}{\chi_{T}}\right)$, where $\upsilon_{P_{A}=0}$ represents the intercept obtained from the linear fit of Raman shift as a function of absorbed power.

### 4.4 Computational Methods

In this work, first‐principles density functional theory (DFT) calculations are carried out using the VASP package with a plane‐wave energy cut‐off of 450 eV. The projector‐augmented wave (PAW) method within the generalized gradient approximation (GGA‐PBE) is employed to describe the Re: {s, d} and S: {p} valence electrons. For the bulk and monolayer 12‐atom unit cell of ReS_2_, the Brillouin zone (BZ) is sampled using a 7 × 7 × 1 Monkhorst–Pack k‐point grid. Structural relaxations are performed until the interatomic forces are reduced below 0.01 eV/Å.

To investigate the harmonic phonon spectra, second‐order interatomic force constants (IFCs) are obtained using density functional perturbation theory (DFPT) in a 3 × 3 × 1 supercell, as implemented in the Phonopy package [59]. The lattice thermal conductivity (κ_*L*_) is then evaluated by explicitly considering phonon–phonon scattering processes within the anharmonic approximation. Anharmonic IFCs are computed by including only three‐phonon interactions, using the Phono3py package [60]. To limit computational cost, phonon–phonon interactions beyond 6 Å are neglected by applying the cut‐off pair method, which is well suited for the distorted trigonal $\left(P\bar{1}\right)$ phase of both bulk and monolayer ReS_2_.

The harmonic and anharmonic IFCs are used as inputs for solving the phonon Boltzmann transport equation (BTE) beyond the relaxation time approximation (RTA) on a dense 35 × 35 × 1 q‐point grid (convergence tests are also verified at finer grids). Within the RTA framework, the lattice thermal conductivity(κ_*L*_) is expressed as:

$$\kappa_{L}=\frac{1}{NV}\sum_{\lambda }\kappa_{L}\left(T\right)=\frac{1}{NV}\sum_{\lambda }C_{\lambda }\upsilon_{\lambda }⊗\upsilon_{\lambda }\tau_{\lambda }$$

where, the sum is taken over all phonon modes λ of the system. Here, *N* and *V* are the number of phonon wavevectors and the unit cell volume, respectively. *C*_λ_and τ_λ_denote the mode‐specific heat capacity and single‐mode relaxation time, while, $\upsilon_{\lambda }=\frac{\partial \omega_{\lambda }}{\partial q}$, represents the phonon group velocity along the lattice directions. To quantify anisotropy in the phonon response, the mode Grüneisen parameter(γ_*i*_) is also calculated, which captures the sensitivity of phonon frequencies to volume changes along different BZ directions. This parameter plays an essential role in understanding the thermal expansion behavior in these systems.

## Author Contributions

**Manab Mandal**: conceptualization, investigation, Writing – original draft, methodology, formal analysis, data curation, Writing – review and editing, visualization. **Prahalad Kanti Barman**: validation, methodology, conceptualization, writing – review and editing, formal analysis, software, writing – original draft. **Susmita Jana**: writing – review and editing, methodology, investigation, software. **Saroj Poudyal**: methodology, formal analysis. **Kaushalya Kumari**: methodology, formal analysis, data curation, software, writing – review and editing. **Sourav Mondal**: methodology, validation, software. **Abhishek Misra**: writing – review and editing, supervision, validation. **Birabar Ranjit Kumar Nanda**: writing – review and editing, validation, supervision. **Pramoda Kumar Nayak**: investigation, funding acquisition, writing – review and editing, validation, supervision, visualization. **Kanikrishnan Sethupathi**: investigation, writing – review and editing, validation, supervision.

## Conflicts of Interest

The authors declare no conflicts of interest.

## Supporting information




**Supporting File**: advs77734‐sup‐0001‐SuppMat.pdf.

## Data Availability

The data that support the findings of this study are available from the corresponding author upon reasonable request.
