# Supplementary material for: Unveiling Anisotropic in‐Plane Thermal Conductivity and Thermal Expansion Coefficient in Low‐Symmetry Few‐Layer ReS2
Source: Adv Sci (Weinh). 2026 Sep 27:e77734. Online ahead of print. doi: 10.1002/advs.77734 (PMC13616395; doi:10.1002/advs.77734)
Supplement: Supplementary file 1 [file ADVS-9999-e77734-s001.pdf]

Supporting Information

Unveiling anisotropic in-plane thermal conductivity and thermal expansion coefficient in low-symmetry few-layer ReS_2

Manab Mandal, Prahalad Kanti Barman, Susmita Jana, Saroj Poudyal, Kaushalya Kumari, Sourav Mondal, Abhishek Misra, Birabar Ranjit Kumar Nanda, Pramoda Kumar Nayak*, Kanikrishnan Sethupathi**

Manab Mandal, Kaushalya Kumari, Sourav Mondal, Kanikrishnan Sethupathi

Low Temperature Physics Laboratory, Department of Physics, Indian Institute of Technology Madras, Chennai 600036, India
E-mail: ksethu@iitm.ac.in

Prahalad Kanti Barman, Saroj Poudyal, Abhishek Misra, Pramoda Kumar Nayak

2D Materials Research and Innovation Group, Department of Physics, Indian Institute of Technology Madras, Chennai 600036, India

Susmita Jana, Birabar Ranjit Kumar Nanda

Center for Atomistic Modelling and Materials Design, Department of Physics, Indian Institute of Technology Madras, Chennai-600036, India.
E-mail: nandab@iitm.ac.in

Pramoda Kumar Nayak

Centre for Nano and Material Sciences, Jain (Deemed-to-be University), Jain Global Campus, Bangalore 562112, India.
E-mail: pramoda.nayak@jainuniversity.ac.in

1 Exfoliated few-layer ReS_2 flakes on an array of 5 µm holes and thickness determination

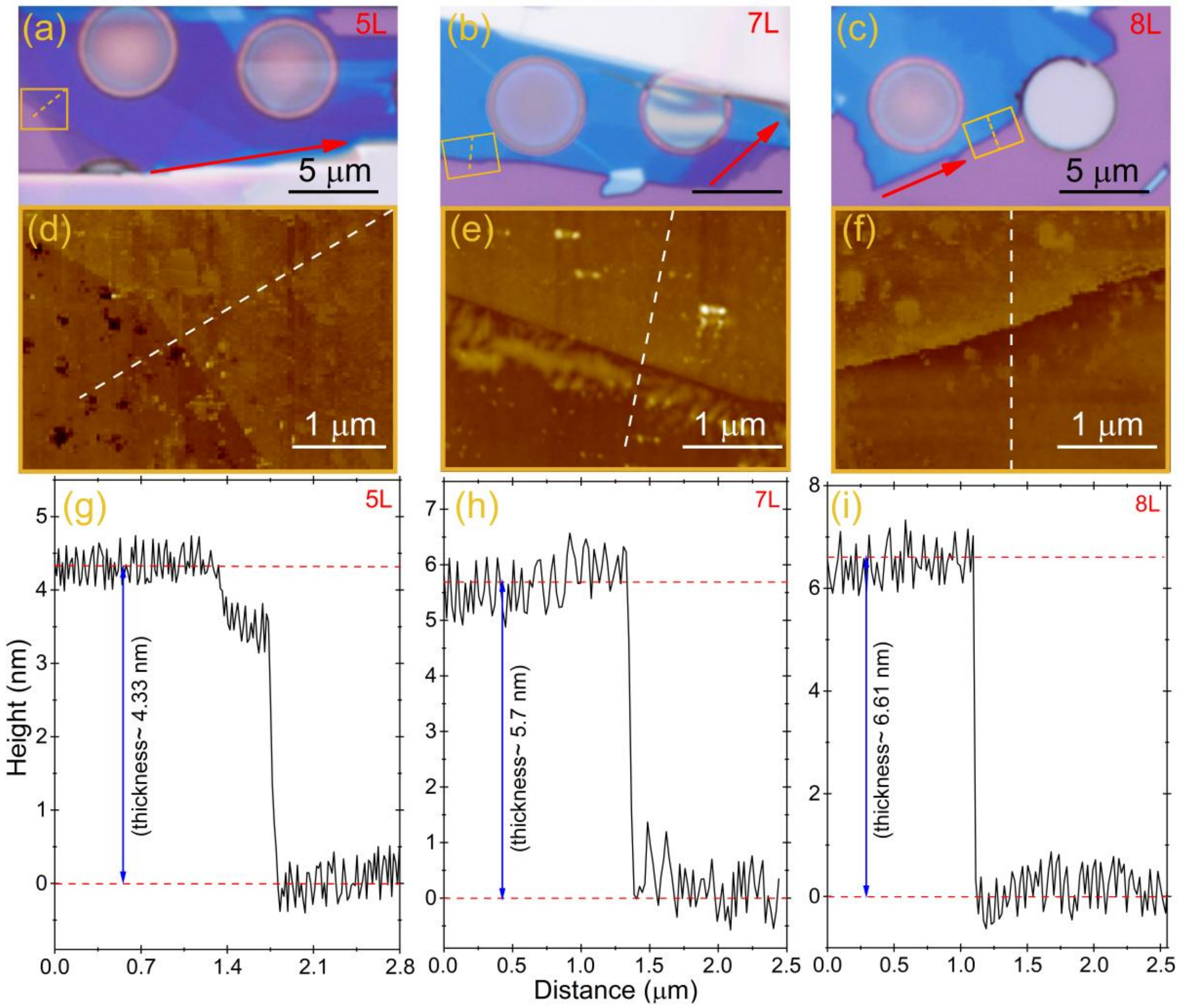

Figure S1. Mechanically exfoliated few-layer ReS_2 and determination of their thickness. (a-c) Optical microscopy images of *5L*, *7L*, and *8L*-ReS_2 flakes suspended over 5 µm holes (scale bar: 5 µm). The red arrows mark the crystallographic '*b*'-axis corresponding to the *Re*-*Re* diamond-chain direction. (d-f) Atomic force microscopy (AFM) images of *5L*, *7L*, and *8L*-ReS_2 flakes (scale bar: 1 µm). (g-i) Present corresponding AFM height profile of *5L*, *7L* and *8L*-ReS_2 flakes, respectively.

2 Raman spectra of few-layer ReS_2

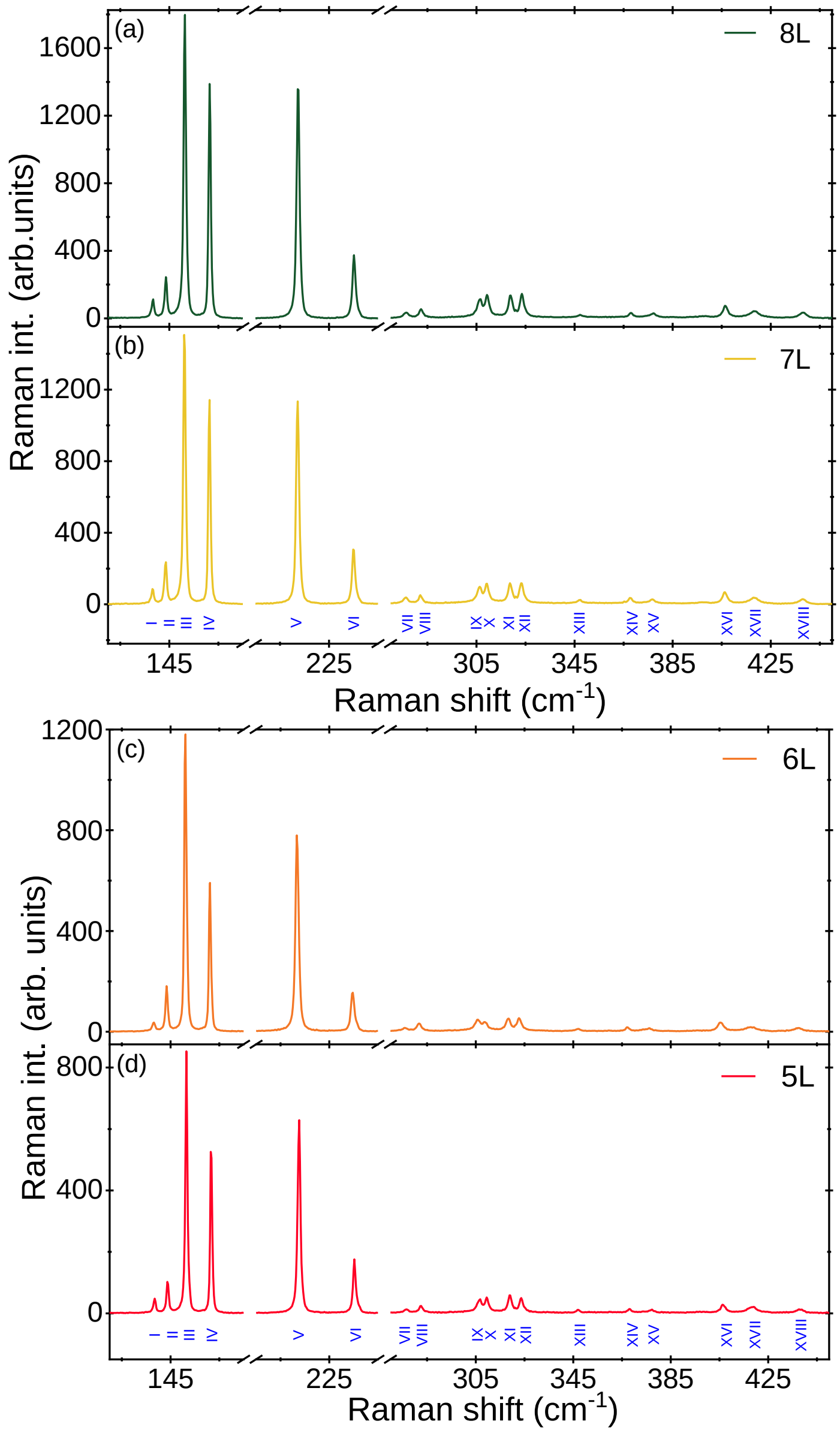

Figure S2. Measured Raman spectra of few-layer ReS_2. (a-d) Layer-dependent Raman spectra of *8L*, *7L*, *6L*, and *5L* ReS_2 under 632.8 nm laser excitation. The Raman-active modes are labeled with Roman numerals I-XVIII, corresponding to the 18 symmetry-allowed vibrational modes.

Table S1: Peak positions as a function of layer number.

Modes (cm^{-1}) / Layer Number	I	II	III	IV	V	VI
5L	138.52	143.70	151.47	161.51	212.66	235.32
6L	138.20	143.38	151.15	161.18	211.69	234.67
7L	138.12	143.62	151.09	161.42	212.22	234.87
8L	138.12	143.61	151.06	161.42	212.23	235.20

3 Note I: Polarized Raman spectra of few-layer ReS_2

This polarization dependence can be analyzed in terms of the Raman tensor for all Raman active modes of the C_i point group as,

$$R = \begin{pmatrix} a & d & e \\ d & b & f \\ e & f & c \end{pmatrix} \tag{S1}$$

where, a, b, c, d, e, and f are constants. The Raman mode intensity can be expressed as a function of the crystal symmetry and polarization geometry as below,

$$I \propto \left| \vec{e}_R . R . \vec{e}_L \right|^2 \tag{S2}$$

where, $\vec{e}_R$ and $\vec{e}_L$ are the unit polarization vectors of the Raman signal and laser polarization, respectively. Herein, the magnitude of the unit vectors, $\vec{e}_R$ and $\vec{e}_L$ are $e_R = (0 \quad 1 \quad 0)$ and $e_L = (\cos\theta \quad \sin\theta \quad 0)$. The resultant intensity distribution can be expressed as,

$$I \propto m\cos^2\theta + \sin 2\theta + \frac{1}{m}\sin^2\theta \tag{S3}$$

where, $d/b = m$. In our observation, all the Raman modes fit well with the above equation.

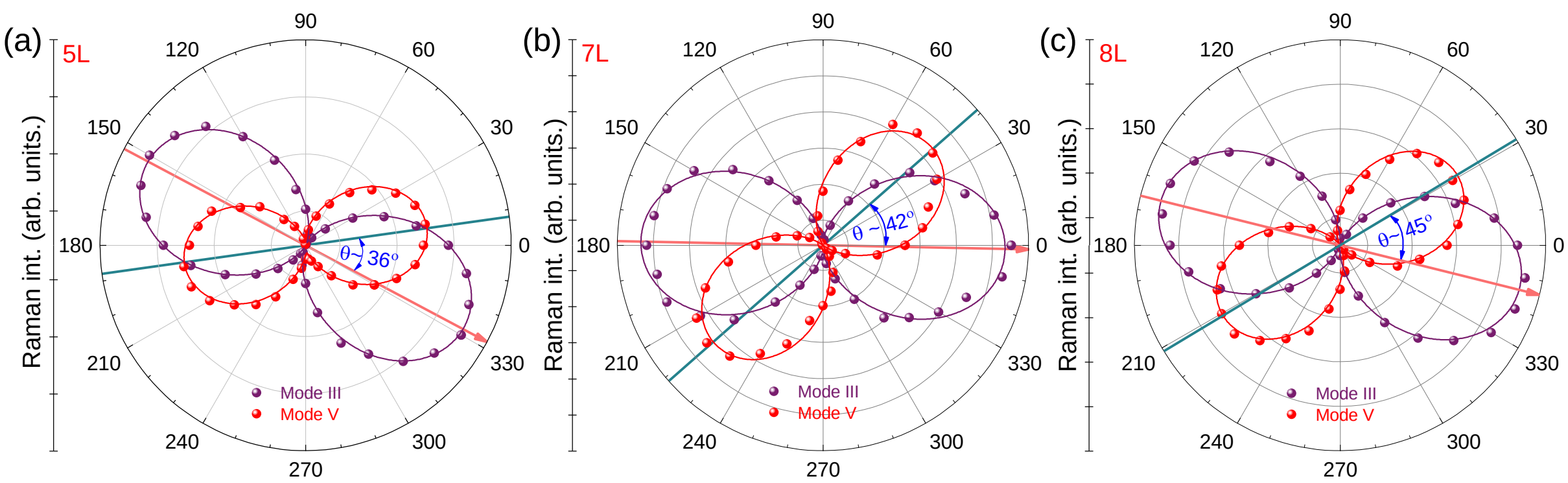

Figure S3. Angle-resolved Raman spectra of few-layer ReS_2. (a-c) Polarization-dependent Raman intensities of modes III (violet spheres) and V (red spheres) in five-layer (*5L*), seven-layer (*7L*), and eight-layer (*8L*) ReS_2. The measurements reveal angular deviations of 36°, 42°, and 45°, respectively, between the directions of maximum intensity.

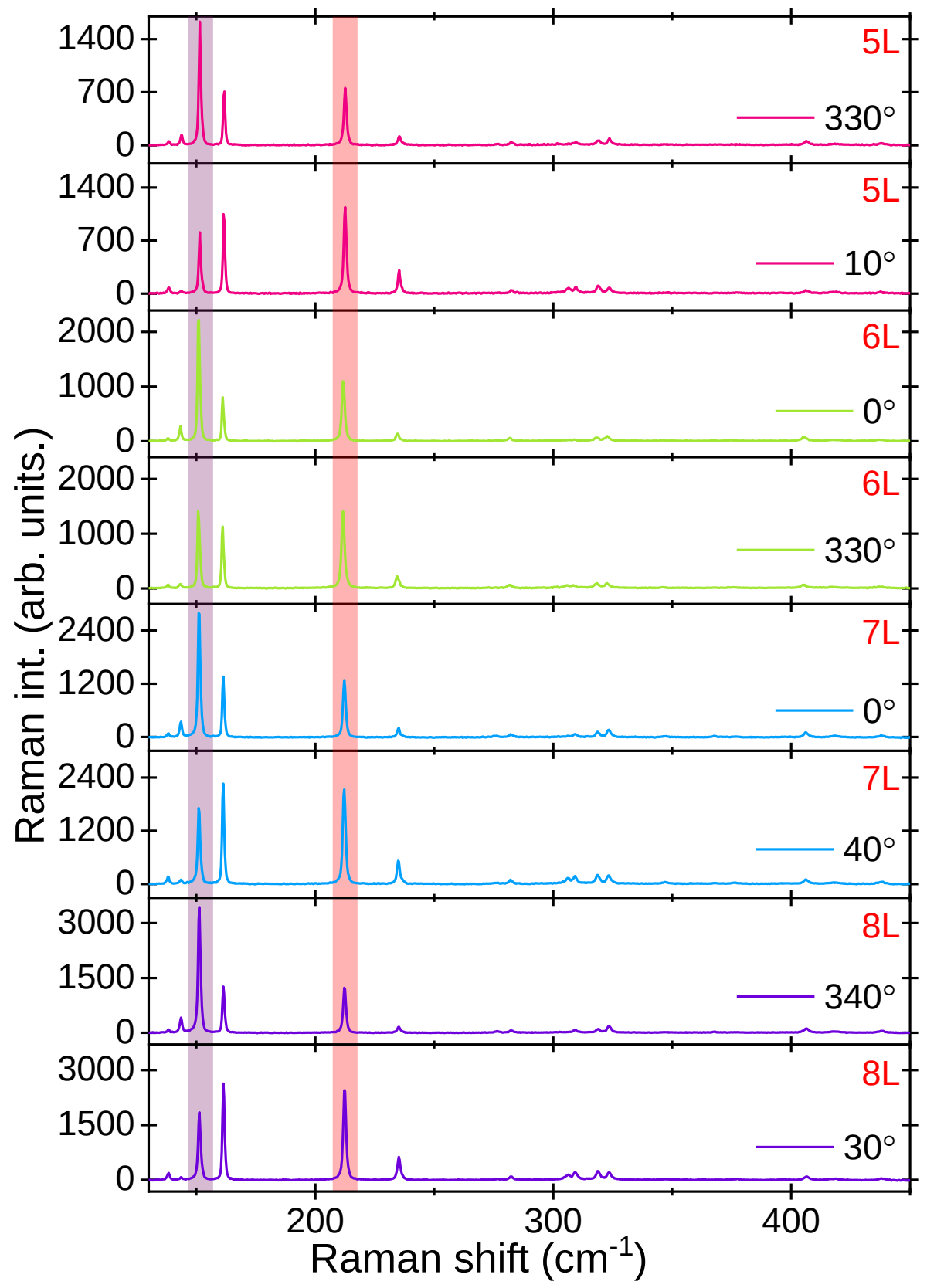

Figure S4. Angle-resolved Raman spectra of few-layer ReS_2 at specific polarization angles corresponding to the maximum intensities of modes III and V.

4 Determination of the laser spot size

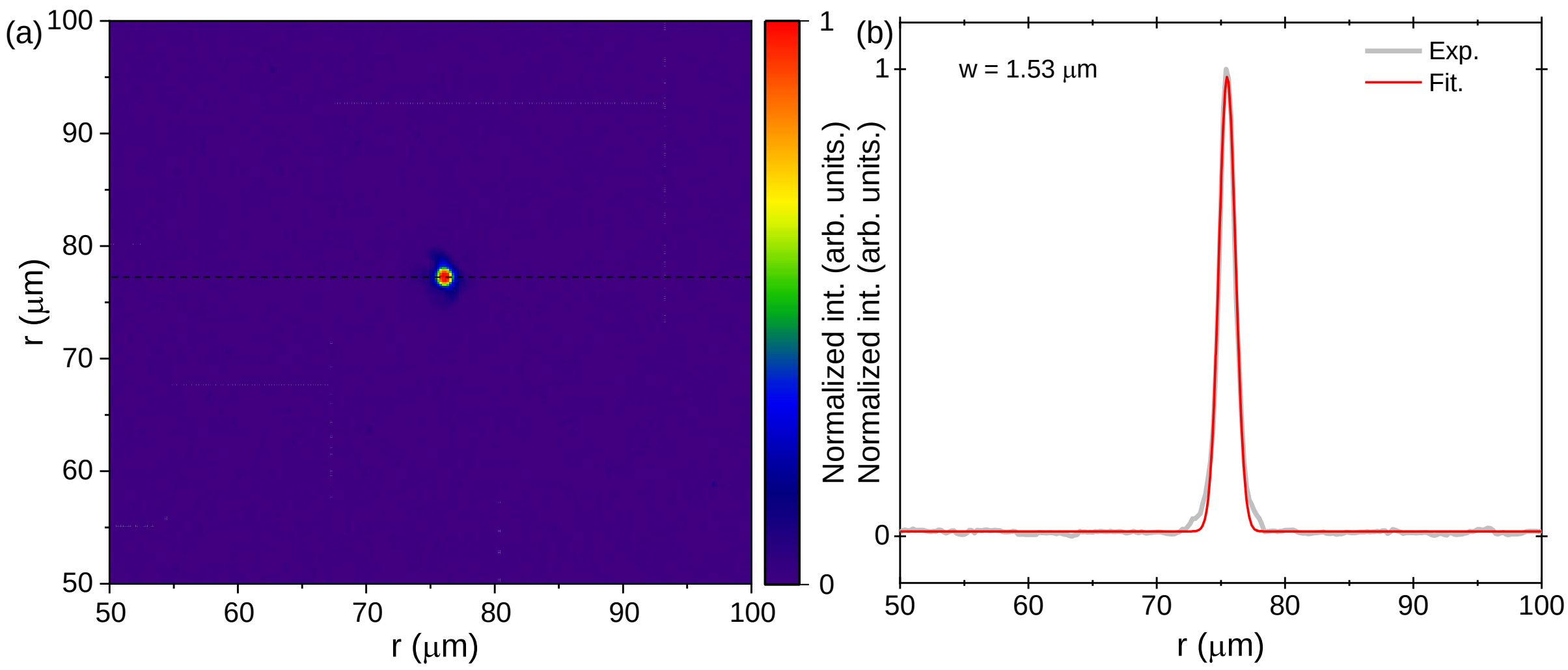

Figure S5. Laser spot size measurement. (a) 2D image of the reflected, tightly focused laser spot. (b) Experimental profile (grey curve) and Gaussian fitting (red curve) of the horizontal 1D cross-section of the laser spot, yielding a spot size of $w = 1.53$ μm.

5 Theoretical calculations of optical absorption along '*a*' and '*b*'-axes

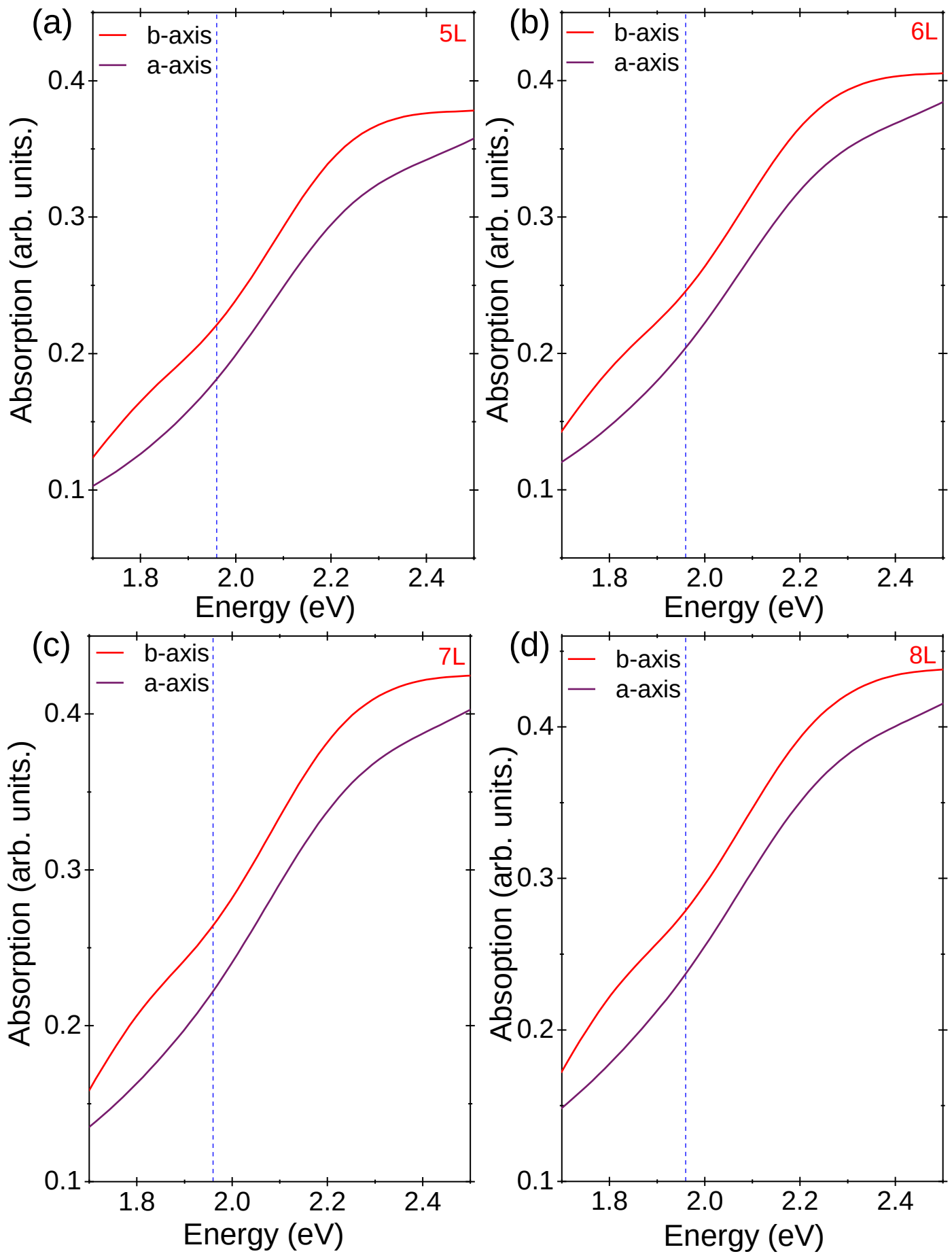

Figure S6. Optical absorption spectra along '*a*' and '*b*'-axes. (a-d) Optical absorption spectra of five-layer (*5L*), six-layer (*6L*), seven-layer (*7L*), and eight-layer (*8L*) ReS_2. Violet and red curves correspond to absorption along the '*a*' and '*b*'-axes, respectively. The dotted vertical line indicates the laser excitation energy (1.96 eV) used throughout the Raman thermometry measurements.

Table S2: A percentage of laser absorption (at 632nm laser excitation) as a function of layer number.

Thickness	Absorption along *a*-axis (%)	Absorption along *b*-axis (%)
5L	18.14	22.11
6L	20.41	24.57
7L	22.23	26.44
8L	23.7	27.89

6 Note II: Finite-Element Method (FEM) simulations of anisotropic heat diffusion

To rigorously analyze the heat-diffusion kinetics and substantiate our analytical model, we performed two-dimensional finite-element method (FEM) simulations of steady-state thermal transport in suspended ReS_2 flakes (1L and 5L). Upon applying a symmetric, circular Gaussian laser heat flux to the highly anisotropic ReS_2 medium, the simulation yields a pronounced elliptical surface temperature profile (ΔT), as illustrated in **Figures S7 a** and **b**. Because thermal energy dissipates preferentially along the high-conductivity direction, the resulting spatial temperature gradients are fundamentally asymmetric; the elongation of the thermal ellipse corresponds to rapid heat conduction along the crystallographic b-axis (governed by κ_b), while the steeper temperature gradient along the minor axis reflects slower heat conduction along the a-axis (governed by κ_a).

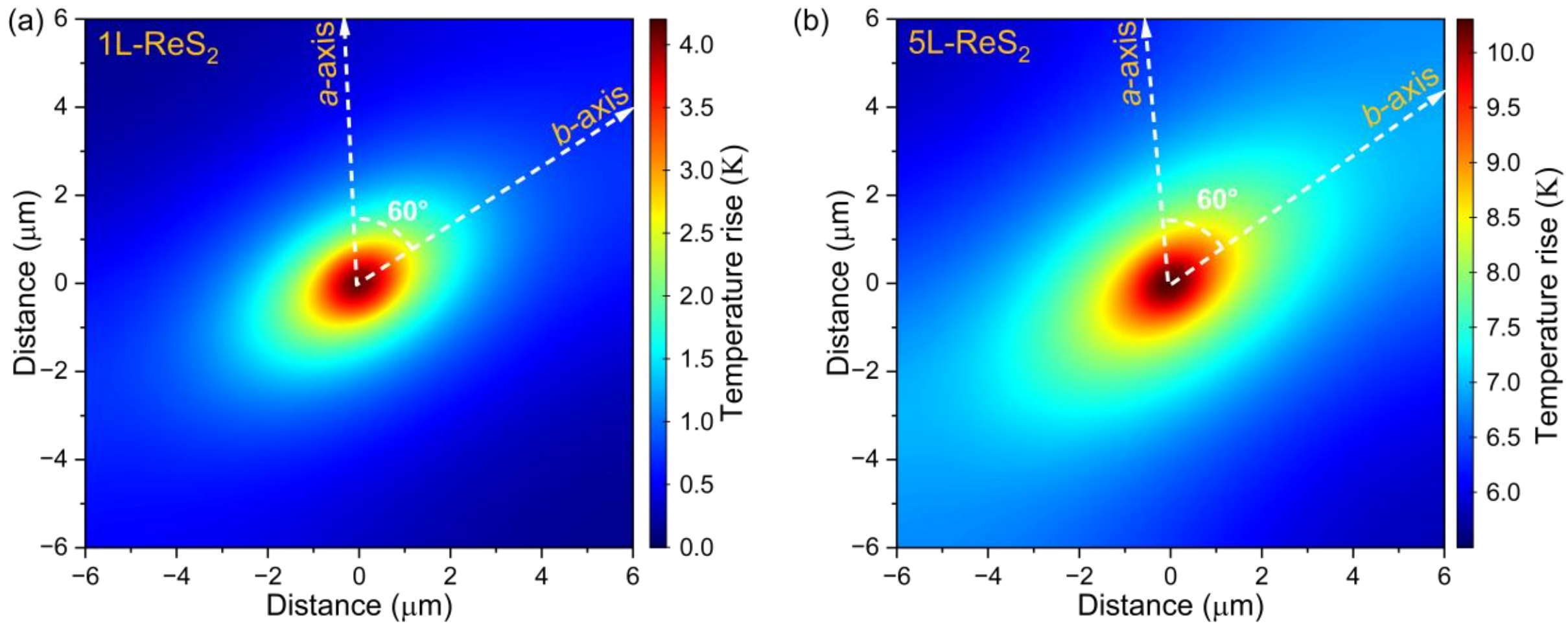

Figure S7. 2D finite-element method (FEM) simulations of steady-state thermal diffusion in suspended (a) *1L* and (b) *5L*-ReS_2.

Given that Raman Modes III and V are directionally polarized along these non-orthogonal crystallographic axes (separated by a 60° crystallographic angle), their respective Raman shifts with respect to the local temperature rise act as independent probes for thermal dissipation along the minor and major axes of this elliptical profile. Our FEM simulations confirm that the local thermal bath beneath the laser excitation spot is directionally heterogeneous, firmly validating our methodology of extracting κ_a and κ_b independently via simultaneous two-mode Raman thermometry without assuming an isotropic medium or relying on a single, homogenized effective thermal conductivity.

7 Power-dependent Raman study on suspended few-layer ReS_2

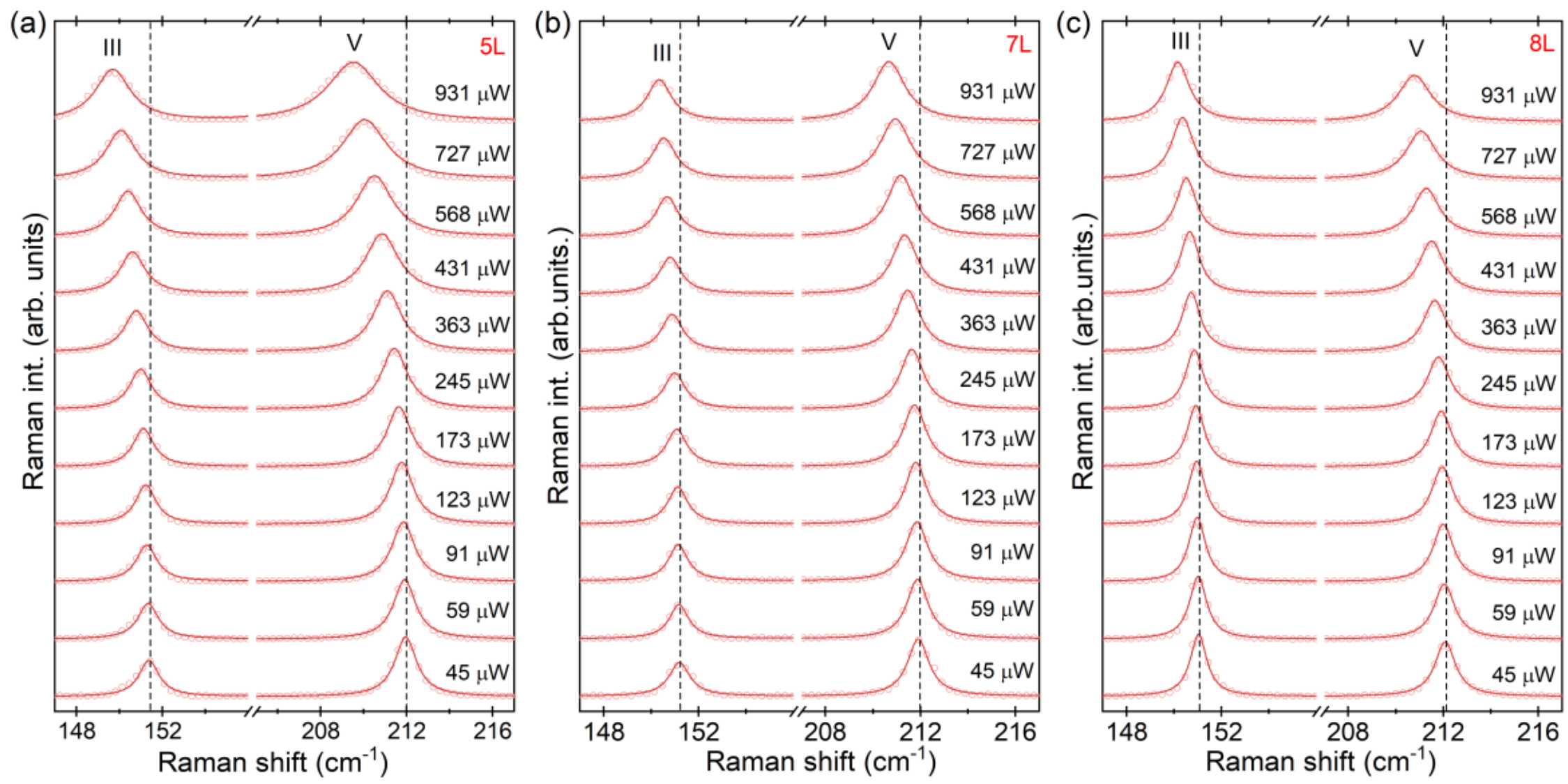

Figure S8. Raman spectra as a function of incident laser power on suspended few-layer ReS_2. (a-c) Room-temperature Raman spectra of a *5L*,*7L*, and *8L*-ReS_2 flake acquired under incident laser powers ranging from 45 μW to 931 μW. Dotted vertical lines indicate the peak positions of Raman modes III and V at an incident laser power of 45 μW.

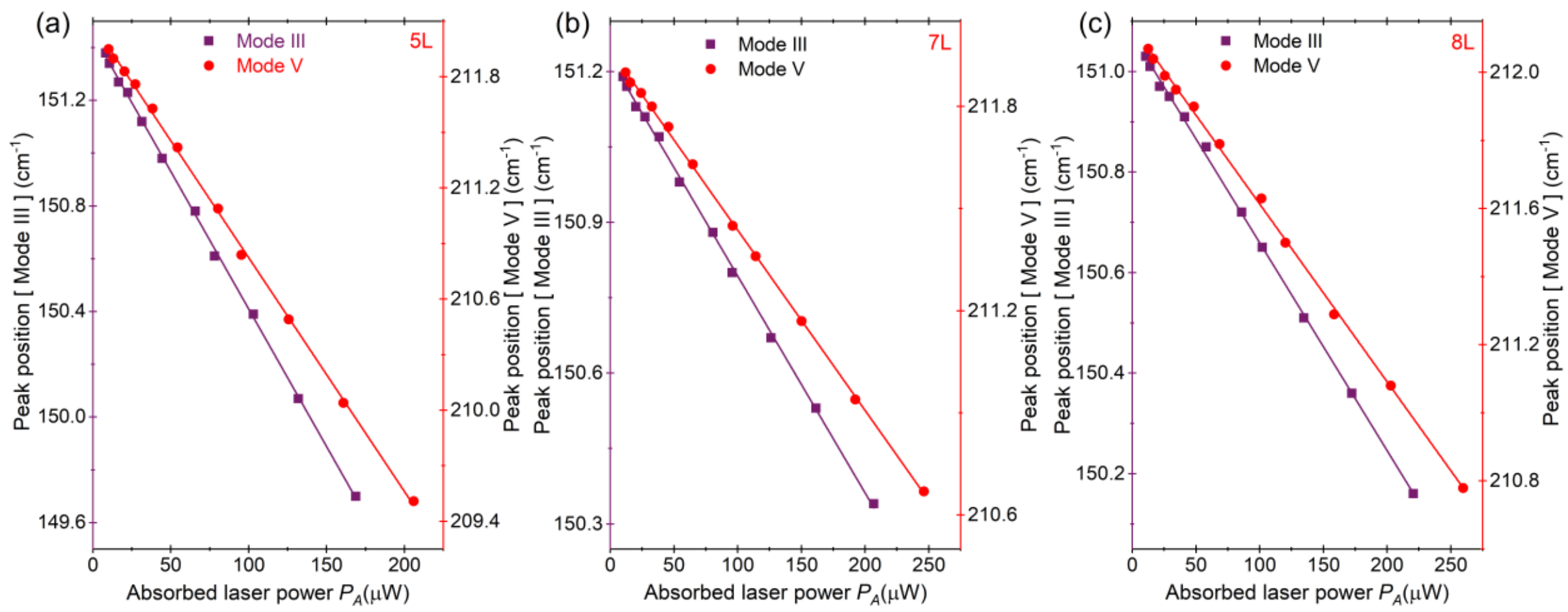

Figure S9. Peak positions of Raman modes (III and V) as a function of absorbed laser power. (a-c) Power-dependent shifts in the peak positions of Raman modes III (violet rectangles) and V (red circles) in suspended five-layer (*5L*), seven-layer (*7L*), and eight-layer (*8L*) ReS_2 flakes, respectively.

8 Power-dependent Raman peak shift of mode III and mode V under polarized (along the '*a*' and '*b*'-axes) and unpolarized conditions for eight-layer (8L) suspended ReS_2

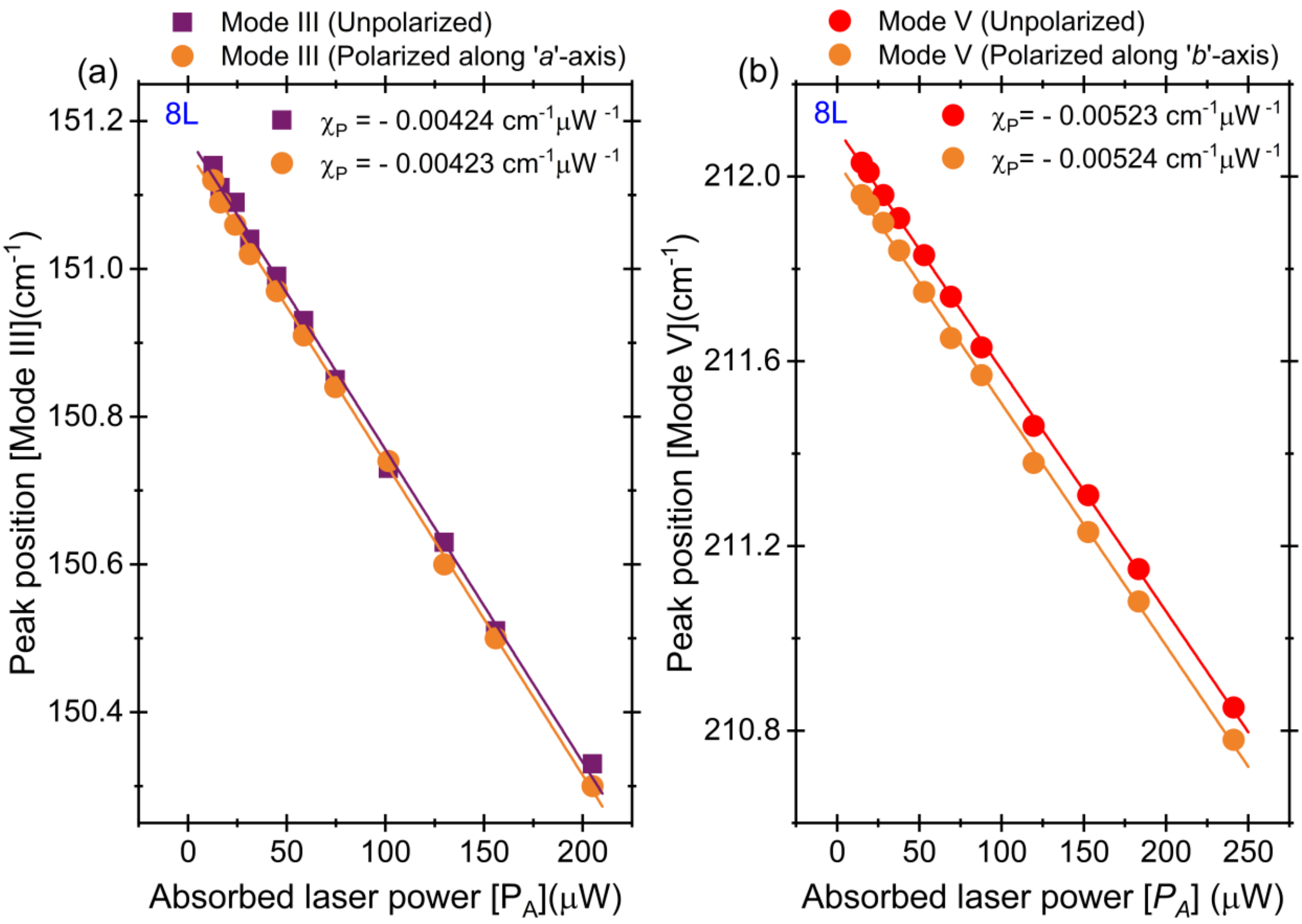

Figure S10. Absorbed laser power-dependent Raman peak shift of mode III and mode V under polarized and unpolarized conditions. (a) Power-dependent shifts in the peak positions of Raman mode III under unpolarized (violet rectangles) and polarized along the '*a*'-axis (yellow circles) conditions. (b) Power-dependent shifts in the peak positions of Raman mode V under unpolarized (red circles) and polarized along '*b*'-axis (yellow circles) conditions.

9 Temperature-dependent Raman study on suspended few-layer ReS_2

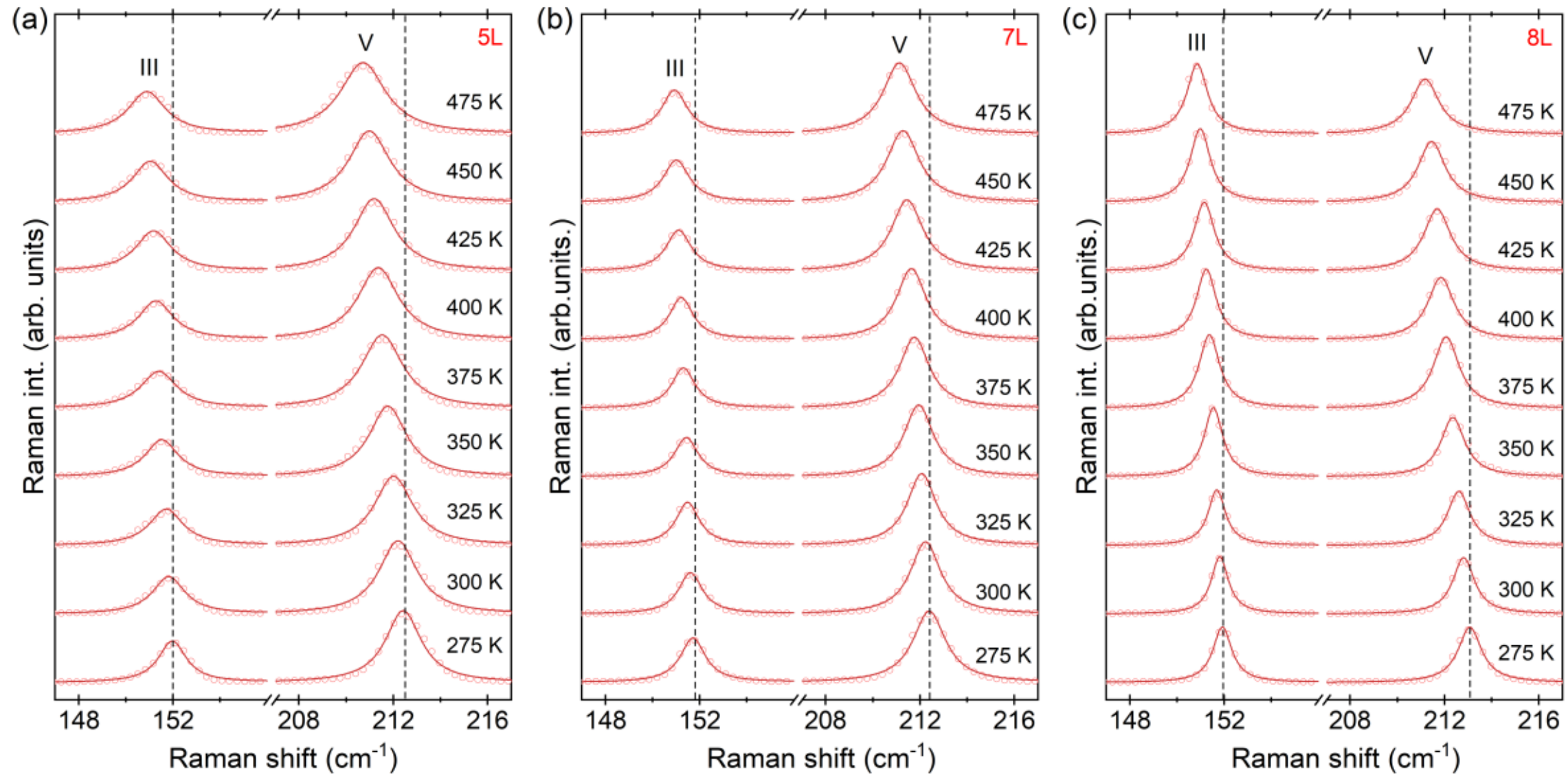

Figure S11. Raman spectra at different temperatures on suspended few-layer ReS_2. (a-c) Raman spectra of the *5L*, *7L*, and *8L* suspended ReS_2 flakes were measured over the temperature range 275 K to 475 K, respectively. The black dotted lines indicate the reference peak positions of modes III and V at 275 K.

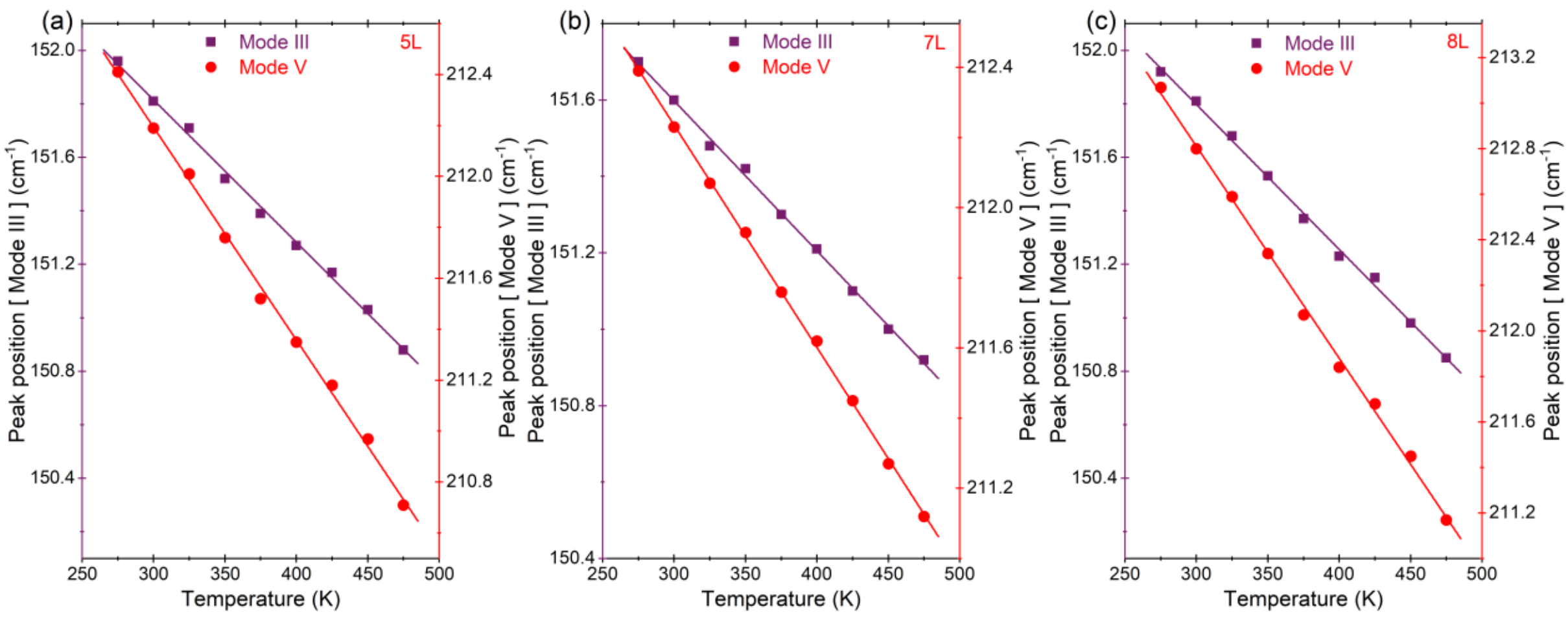

Figure S12. Peak positions of Raman modes (III and V) as a function of temperature. (a-c) Temperature-dependent shifts in the peak positions of Raman modes III (violet rectangles) and V (red circles) in suspended five-layer (*5L*), seven-layer (*7L*), and eight-layer (*8L*) ReS_2 flakes, respectively.

10 Table S3. Layer-dependent first-order power and temperature coefficients of suspended few-layer ReS_2 along the '*a*'- and '*b*'-axes

Layer number (thickness[nm])	$(\chi_P^{Suspended})\times10^{-2}\ \text{cm}^{-1}\mu\text{W}^{-1}$		$(\chi_T^{Suspended})\times10^{-2}\ \text{cm}^{-1}\text{K}^{-1}$	
	'*a*'-axis	'*b*'-axis	'*a*'-axis	'*b*'-axis
5L (4.33 nm)	-1.042±0.008	-1.262±0.011	-0.533±0.011	-0.835±0.014
6L (4.91 nm)	-0.650±0.007	-0.900±0.007	-0.455±0.009	-0.815±0.015
7L (5.7 nm)	-0.434±0.004	-0.529±0.003	-0.393±0.006	-0.634±0.005
8L (6.61 nm)	-0.413±0.004	-0.522±0.005	-0.542±0.010	-0.931±0.016

11 Note III: Uncertainty Analysis of the estimated thermal conductivities

The total uncertainty in the extracted thermal conductivity κ was determined by propagating both the statistical fitting errors and the systematic experimental uncertainties. The fractional uncertainty was calculated using the standard root-sum-square formula:

$$\frac{\Delta\kappa}{\kappa}=\sqrt{\left(\frac{\Delta P_{abs}}{P_{abs}}\right)^2+\left(\frac{\Delta r_0}{r_0}\right)^2+\left(\frac{\Delta R}{R}\right)^2+\left(\frac{\Delta t}{t}\right)^2+\left(\frac{\Delta(\partial\omega/\partial T)}{(\partial\omega/\partial T)}\right)^2+\left(\frac{\Delta(\partial\omega/\partial P)}{(\partial\omega/\partial P)}\right)^2+\left(\frac{\Delta T}{T}\right)^2}$$

The individual contributions were estimated as follows:

- **Laser spot size:** $(\Delta r_0/r_0)$= (0.02/1.53) ~ 0.01307
- **Absorbed power:** $(\Delta P_{abs}/P_{abs})$ = (0.51/10) ~ 0.051
- **Temperature calibration:** $(\Delta T/T)$ = (2/300) ~ 0.0067
- **Hole radius :** $(\Delta R/R)$ = (0.17/5) ~ 0.034
- **Thickness:**

	5L	6L	7L	8L
$(\Delta t/t)$	(0.11/4.33) ~ 0.0254	(0.1/4.91) ~ 0.0203	(0.12/5.7) ~ 0.0211	(0.13/6.61) ~ 0.0196

- **Temperature coefficient:**

	5L	6L	7L	8L
$\left(\frac{\Delta(\partial\omega/\partial T)}{(\partial\omega/\partial T)}\right)_a$	~ 0.0077	~ 0.0107	~ 0.0092	~ 0.0096
$\left(\frac{\Delta(\partial\omega/\partial T)}{(\partial\omega/\partial T)}\right)_b$	~ 0.0087	~ 0.0078	~ 0.0057	~ 0.0097

- **Power coefficients:**

	5L	6L	7L	8L
$\left(\frac{\Delta(\partial\omega/\partial P)}{(\partial\omega/\partial P)}\right)_a$	~ 0.0206	~ 0.0197	~ 0.0152	~ 0.0184
$\left(\frac{\Delta(\partial\omega/\partial P)}{(\partial\omega/\partial P)}\right)_b$	~ 0.0167	~ 0.0184	~ 0.0089	~ 0.0171

Total estimated uncertainty:

	5L	6L	7L	8L
$\left(\frac{\Delta\kappa}{\kappa}\right)_a$	~ 7.14 %	~ 6.99 %	~ 6.88 %	~ 6.92 %
$\left(\frac{\Delta\kappa}{\kappa}\right)_b$	~ 7.05 %	~ 6.91 %	~ 6.73 %	~ 6.89 %

12 Table S4. Layer-dependent in-plane thermal conductivities of ReS_2 along the '*a*' and '*b*'-axes, together with their anisotropy ratios

Layer number (thickness[nm])	Thermal conductivity along '*a*'-axis (κ_a) $Wm^{-1}K^{-1}$	Thermal conductivity along '*b*'-axis (κ_b) $Wm^{-1}K^{-1}$	Anisotropic Ratio
5L (4.33 nm)	22.25 ± 1.59	28.78 ± 2.03	1.29
6L (4.91 nm)	26.65 ± 1.86	34.74 ± 2.40	1.30
7L (5.7 nm)	29.92 ± 2.06	39.61 ± 2.66	1.32
8L (6.61 nm)	37.40 ± 2.59	50.83 ± 3.50	1.36

13 Determination of temperature rise as a function of absorbed laser power

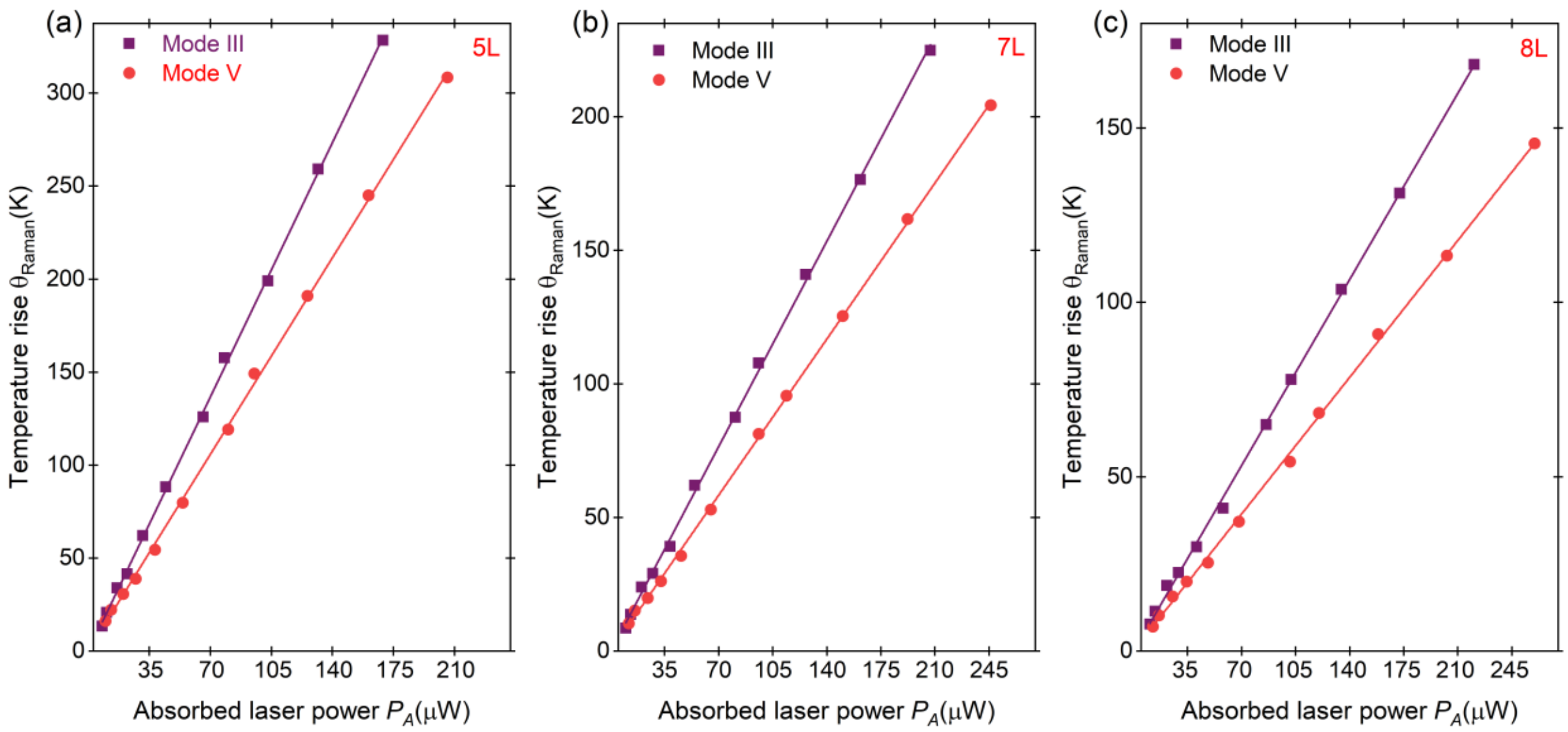

Figure S13. Temperature rise of a few-layer suspended ReS_2 as a function of absorbed laser power. (a-c) Absorbed power (P_A)dependent temperature rise (θ_{Raman}) of the *5L*, *7L*, and *8L*-ReS_2 flakes along a and b-axes transport directions, respectively. The lines represent linear fits to the data.

14 Phonon dispersion and PhDOS of monolayer and bulk ReS_2

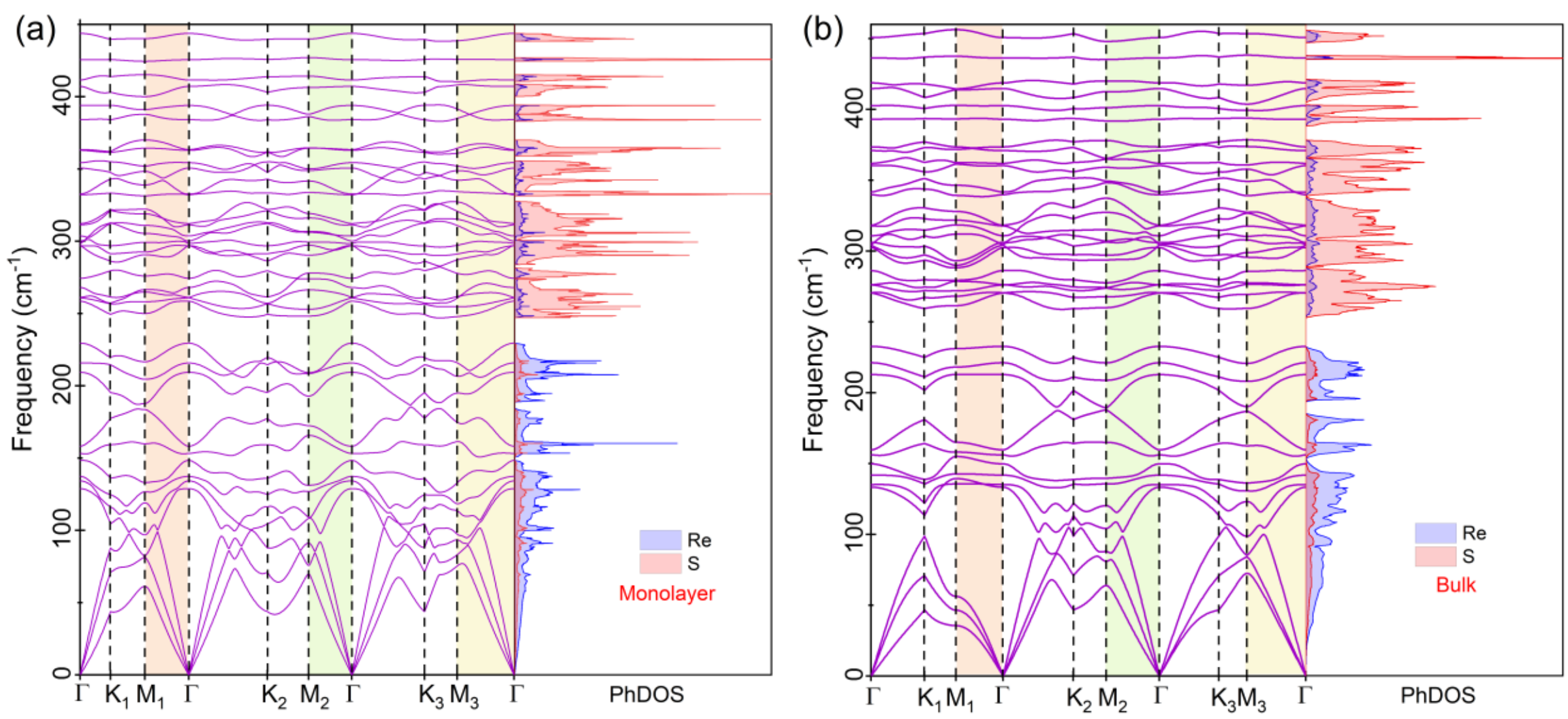

Figure S14. Calculated phonon dispersions and PhDOS of monolayer and bulk ReS_2. Phonon dispersion (left panel) and PhDOS (right panel) for (a) Monolayer, (b) Bulk ReS_2 stacked, respectively.

15 Average phonon group velocities along the Γ-M$_1$ and Γ-M$_2$ paths for *2L* to *5L*-ReS$_2$.

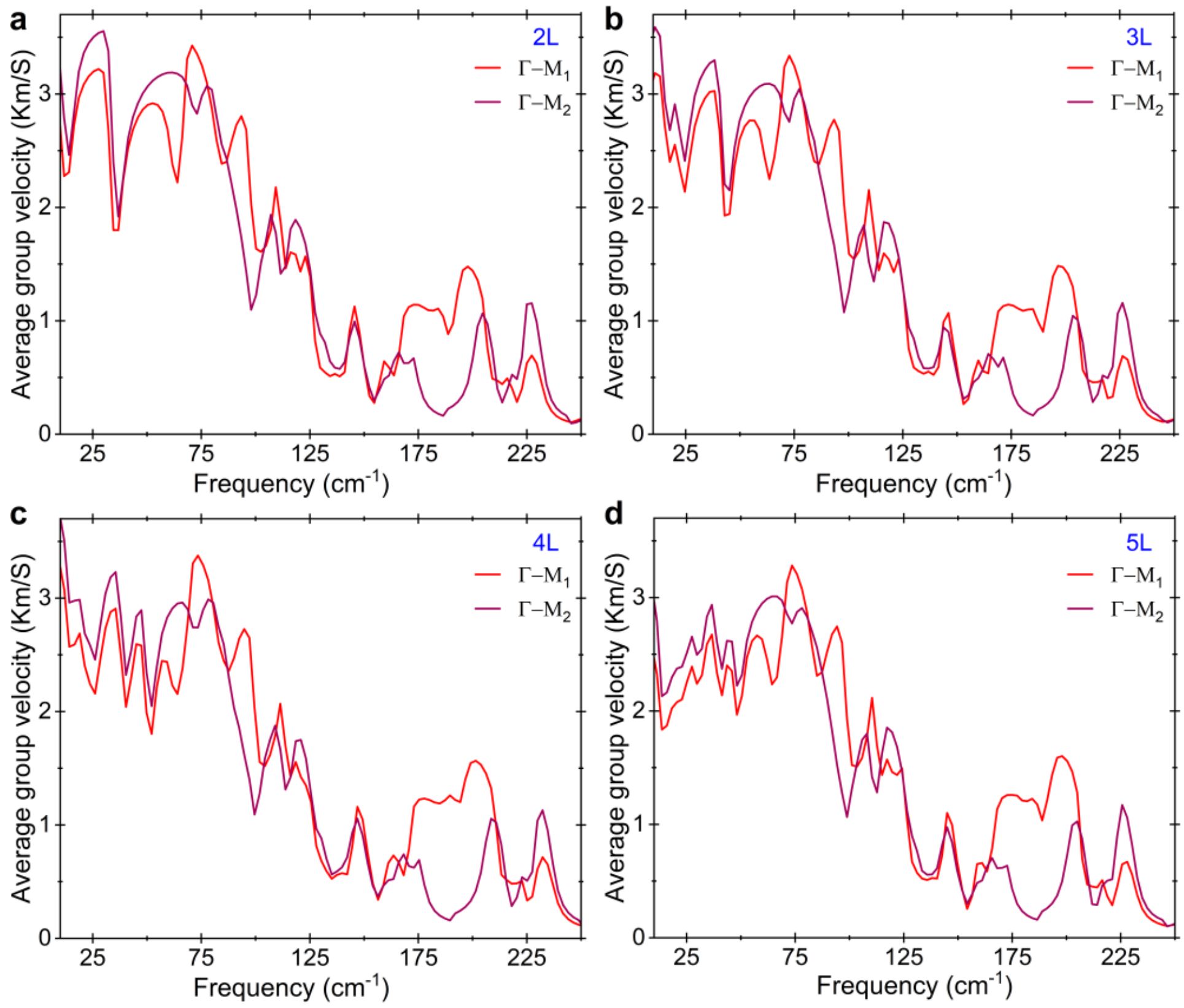

Figure S15. Average phonon group velocities as a function of frequency for *2L* to *5L*-ReS$_2$. (a-d) Mode-dependent average phonon group velocities along the considered crystallographic directions (Γ-M$_1$ and Γ-M$_2$).

16 Mode Grüneisen parameters along the Γ-M$_1$ and Γ-M$_2$ paths for 1L and bulk ReS$_2$.

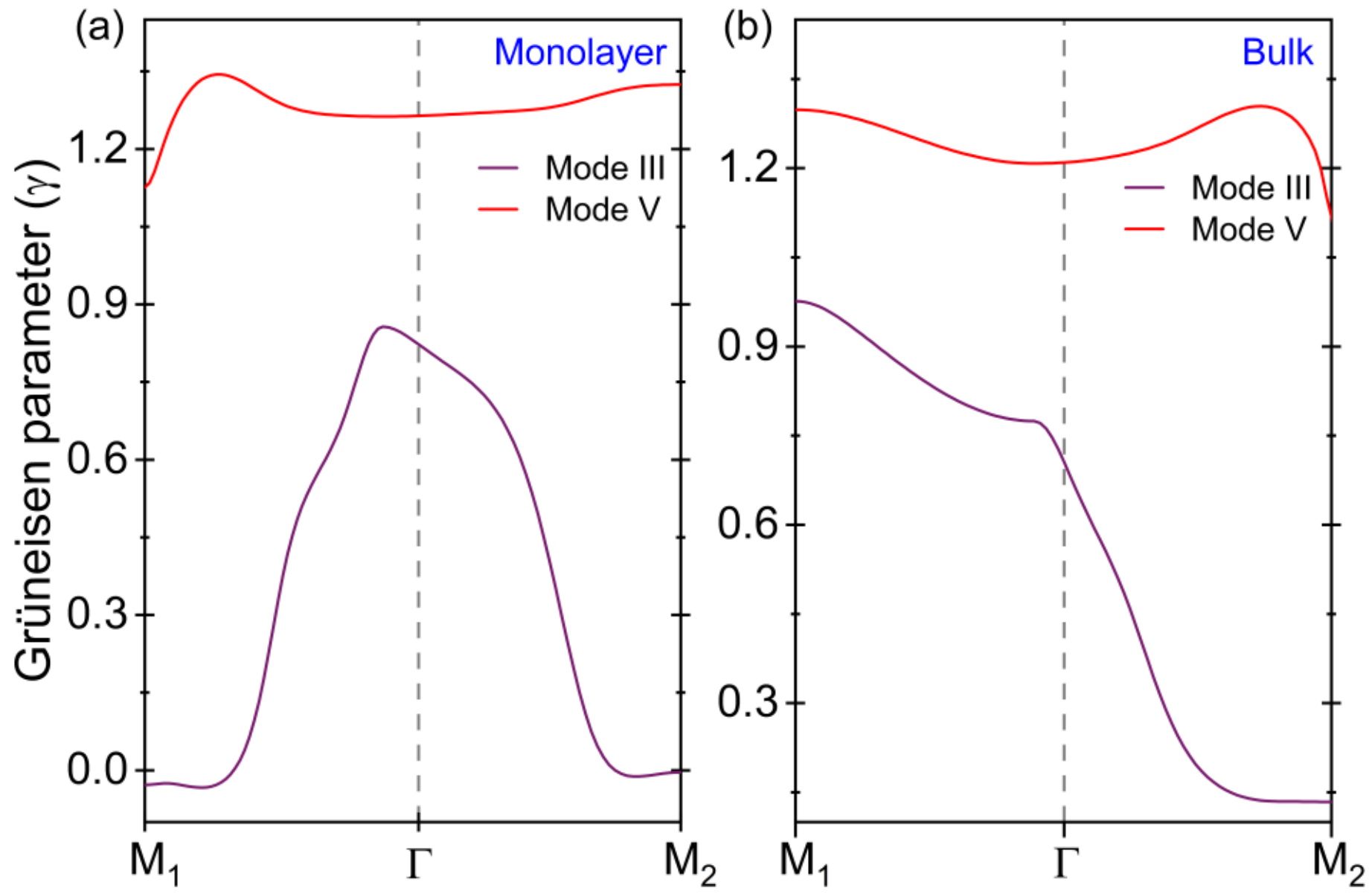

Figure S16. Grüneisen parameter for modes III and V along the opted BZ path. (a-b) Grüneisen parameters of monolayer and bulk ReS2 along the Γ-M$_1$ and Γ-M$_2$ paths, respectively.

17 Note IV: Thermal Expansion Coefficient (TEC) in few-layer ReS_2

To understand the phonon frequency shift with temperature and stress, we have performed a theoretical analysis using group theory. The lattice structure of layered ReS_2 belongs to the C_i (triclinic) point group. According to the factor group analysis, the Raman active optical phonon modes at the Γ point is represented by $\Gamma_{ph} = 18A_g + 18A_u$, where $18A_g$ modes are Raman active. As the mode A_u is forbidden in the backscattering configuration, we studied the irreducible representation $\Gamma_{ph} = 18A_g$ in the following analysis.

According to the perturbation theory, the shift of phonon frequency (vibrational energy level) originates from the temperature rise or stress-induced strain, which perturbs the crystal potential. We considered the first-order perturbation and formulated the strain-induced linear deformation potential,

$$D = \sum D_{ij}\varepsilon_{ij} \qquad i, j = x, y, z \tag{S1}$$

where $\{\varepsilon_{ij}\}$ and $\{D_{ij}\}$ are the symmetric second-rank tensors representing the strain perturbation and the corresponding deformation potential, respectively. So, the total deformation potential due to strain and temperature rise should be $D_{\text{total}} = \sum D_{ij}\varepsilon_{ij} + D^{ph}\Delta T$, where D^{ph} is the temperature-induced perturbation term, which is independent of strain, primarily originates from phonon-phonon interactions.[1] The induced strain ε_{ij} is related to the stress and temperature through the constitutive relation, which can be expressed using the Voigt notation as follows,

$$\varepsilon_i = S_{ij}\sigma_j + \alpha_i \Delta T \tag{S2}$$

where $\{S_{ij}\}$ is the elastic susceptibility tensor of ReS_2, which has the form of,

$$\{S_{ij}\} = \begin{pmatrix} S_{11} & S_{12} & S_{13} & S_{14} & S_{15} & S_{16} \\ S_{21} & S_{22} & S_{23} & S_{24} & S_{25} & S_{26} \\ S_{31} & S_{32} & S_{33} & S_{34} & S_{35} & S_{36} \\ S_{41} & S_{42} & S_{43} & S_{44} & S_{45} & S_{46} \\ S_{51} & S_{52} & S_{53} & S_{54} & S_{55} & S_{56} \\ S_{61} & S_{62} & S_{63} & S_{64} & S_{65} & S_{66} \end{pmatrix} \tag{S3}$$

Using the character table of C_i group (Table S4)[2] the deformation potential can be formulated as,

$$D = D_{xx}\varepsilon_{xx} + D_{yy}\varepsilon_{yy} + D_{zz}\varepsilon_{zz} + 2D_{xy}\varepsilon_{xy} + 2D_{yz}\varepsilon_{yz} + 2D_{zx}\varepsilon_{zx} \tag{S4}$$

It can be seen that the D_{xx}, D_{yy}, D_{zz}, D_{xy}, D_{yz}, and D_{zx} all the terms belong to A_g. Accordingly, the perturbation potential D can be represented by $\Gamma_D = A_g + A_u$.

Table S5: Character table for group C_i=S_2 (triclinic)

$S_2(\bar{1})$			E	i
$x^2, y^2, z^2, xy, yz, zx$	R_x, R_y, R_z	A_g	1	1
	x, y, z	A_u	1	-1

Also, from the perturbation theory, the effect of the deformation potential on the phonon frequency shift can be determined by calculating the matrix element $\langle \Gamma_{ph} | D | \Gamma_{ph} \rangle$. We have obtained the explicit form of $\langle \Gamma_{ph} | D | \Gamma_{ph} \rangle$ by plugging equation S4 for D and analyzed the phonon frequency shift of the A_g mode, which can be expressed as,

$$\Delta\omega^{A_g} = \frac{1}{\hbar} \langle A_g | D | A_g \rangle \tag{S5}$$

where, $\Delta\omega^{A_g}$ is the change in phonon frequency of A_g modes, and $\hbar$ is the reduced Planck's constant. Although in a triclinic (C_i) crystal all six strain components are allowed (since all the quadratic forms are A_g under inversion), when we compute the expectation value in an A_g phonon state, the only contributions that survive are those that transform as A_g. This is a direct consequence of the selection rule:

$$\langle A_g | D | A_g \rangle \neq 0 \text{ ; only if } A_g \otimes \Gamma_D \otimes A_g \supset A_g \tag{S6}$$

Since the analysis is confined to the 2D limit, deformation potential terms associated with out-of-plane components (D_{zz}, D_{yz}, and D_{zx}) are neglected. Therefore, the deformation potential becomes,

$$D = D_{xx}\varepsilon_{xx} + D_{yy}\varepsilon_{yy} + 2D_{xy}\varepsilon_{xy} \tag{S7}$$

The interaction term between the in-plane x and y components (D_{xy}) is neglected in the deformation potential analysis due to weak coupling, so the deformation potential becomes

$$D = D_{xx}\varepsilon_{xx} + D_{yy}\varepsilon_{yy} \tag{S8}$$

Therefore, the change in phonon frequency due to strain perturbation $\{\varepsilon_{ij}\}$ is given by,

$$\Delta\omega^{A_g} = \frac{1}{\hbar}\left\langle A_g \left| D \right| A_g \right\rangle = a^{A_g}\varepsilon_{xx} + b^{A_g}\varepsilon_{yy} \tag{S9}$$

where, $a^{A_g} = \frac{1}{\hbar}\left\langle A_g \left| D_{xx} \right| A_g \right\rangle$ and $b^{A_g} = \frac{1}{\hbar}\left\langle A_g \left| D_{yy} \right| A_g \right\rangle$ are the phonon deformation potential constants for A_g modes. In equation S8, the normal stress components ε_{xx} and ε_{yy} contribute to the change in phonon frequency along the '*a*'-axis and '*b*'-axis, respectively, for a few-layer ReS_2. Additionally, the constitutive relation indicates,

$$\begin{aligned}\varepsilon_{xx} &= S_{11}\sigma_{xx} + S_{12}\sigma_{yy} + \alpha_x\Delta T \\ \varepsilon_{yy} &= S_{21}\sigma_{xx} + S_{22}\sigma_{yy} + \alpha_y\Delta T\end{aligned} \tag{S10}$$

Therefore, we obtain the stress and temperature-dependent phonon frequency shift by plugging Eq. S10 into Eq. S9,

$$\Delta\omega^{A_g} = a^{A_g}(S_{11}\sigma_{xx} + S_{12}\sigma_{yy} + \alpha_x\Delta T) + b^{A_g}(S_{21}\sigma_{xx} + S_{22}\sigma_{yy} + \alpha_y\Delta T) \tag{S11}$$

Since ReS_2 is an anisotropic material, it shows in-plane anisotropic (mechanical, electrical, optical, and thermal) properties along the '*a*'-axis and '*b*'-axis. So, from Eq. S11, we can write the stress and temperature-dependent phonon frequency shift along the '*a*'-axis and '*b*'-axis, respectively, as,

$$\begin{aligned}\Delta\omega_a^{A_g} &= K^a\sigma_a + A^a\Delta T \\ \Delta\omega_b^{A_g} &= K^b\sigma_b + A^b\Delta T\end{aligned} \tag{S12}$$

where $K^b = (a^{A_g}S_{11} + b^{A_g}S_{21})$ and $K^a = (a^{A_g}S_{12} + b^{A_g}S_{22})$ are the stress coefficients along the '*b*'-axis and '*a*'-axis, respectively. $A^b = a^{A_g}\alpha_x$ and $A^a = b^{A_g}\alpha_y$ are the temperature coefficients along the '*b*'-axis and '*a*'-axis, respectively. The strain-stress relations of the flakes supported by SiO_2(290 nm)/Si substrate are expressed as,

$$\varepsilon_{SiO_2}^{a,b} = \alpha^{a,b}\Delta T + \frac{1-\nu}{E_{a,b}}\sigma_{a,b} = \alpha_{SiO_2}\Delta T \tag{S13}$$

where, $\varepsilon_{SiO_2}^{a,b}$ is the in-plane (along either a or b-axis) thermal strain within the ReS_2 flakes on the SiO_2(290 nm)/Si substrate. $\alpha^{a,b}$ and α_{SiO_2} are the in-plane TEC of ReS_2 flakes (along either '*a*' or '*b*'-axis) and SiO_2, respectively. ν represents the Poisson ratio of ReS_2 and E_a and E_b are the Young's modulus along '*a*' and '*b*'-axes, respectively. So, the in-plane (along either '*a*' or '*b*'-axis) thermal stress can be expressed as,

$$\sigma_{a,b} = \frac{E_{a,b}(\alpha_{SiO_2} - \alpha^{a,b})}{1-\nu}\Delta T \quad \text{(S15)}$$

By substituting the Eq. S15 in Eq. S12, we obtained the temperature-dependent frequency shift along the '*a*'-axis and '*b*'-axis as,

$$\begin{aligned} \Delta\omega^a_{SiO_2} &= K^a \frac{(\alpha^a_{SiO_2} - \alpha^a)E_a}{1-\nu}\Delta T + A^a\Delta T \\ \Delta\omega^b_{SiO_2} &= K^b \frac{(\alpha^b_{SiO_2} - \alpha^b)E_b}{1-\nu}\Delta T + A^b\Delta T \end{aligned} \quad \text{(S16)}$$

where, $\Delta\omega^a_{SiO_2}$ and $\Delta\omega^b_{SiO_2}$ are the temperature-dependent frequency shifts of substrate (Si/SiO_2) supported ReS_2 flakes along '*a*'-axis and '*b*'-axis, respectively. From Eqs. 16, we can determine the substrate-supported temperature coefficient of few-layer ReS_2 along the '*a*'-axis and '*b*'-axis, which can be experimentally determined,

$$\begin{aligned} A^a_{SiO_2} &= K^a \frac{(\alpha^a_{SiO_2} - \alpha^a)}{1-\nu}E_a + A^a \\ A^b_{SiO_2} &= K^b \frac{(\alpha^b_{SiO_2} - \alpha^b)}{1-\nu}E_b + A^b \end{aligned} \quad \text{(S17)}$$

The temperature coefficients $A^a_{SiO_2}, A^b_{SiO_2}, A^a, \text{and } A^b$ can be measured from the temperature-dependent Raman experiments. We have expressed the unknown thermal expansion coefficient along the '*a*'-axis and '*b*'-axis related term as a function of temperature coefficients as,

$$\alpha^a = \alpha_{SiO_2} - \frac{1-\nu}{E_a K^a}(A^a_{SiO_2} - A^a) \quad \text{(S18)}$$

and,

$$\alpha^b = \alpha_{SiO_2} - \frac{1-\nu}{E_b K^b}(A^b_{SiO_2} - A^b) \quad \text{(S19)}$$

where α^a and α^b are the thermal expansion coefficients along '*a*'-axis and '*b*'-axis in few layer ReS_2.

18 Note V: Investigating stress-free and no-slip conditions in few-layer ReS_2 flakes

In the manuscript, we have discussed that the suspended region of the few-layer ReS_2 flake over the 5 µm diameter hole is subjected to nearly stress-free conditions, whereas the ReS_2 flake supported on the SiO_2(290 nm)/Si substrate is subjected to a no-slip condition at the interface. In this section, we present a detailed assessment of these conditions to elucidate the mechanical state and strain distribution within the flake in each configuration.

The assumption of a stress-free state in a suspended thin flake has been experimentally verified by Luo *et al.*[3] and subsequently utilized in the determination of thermal expansion coefficients by Bao *et al.*[4] and Zhang *et al.*[5] Here, we present a detailed justification for adopting these assumptions. Since the thermal expansion coefficient along the '*a*'-axis and '*b*'-axis ($\alpha_{5L}^{a} \approx 4.19\times10^{-6}K^{-1}$ and $\alpha_{5L}^{b} \approx 6.37\times10^{-6}K^{-1}$) of few-layer ReS_2 is larger than that of silicon dioxide ($\alpha_{SiO_2} \approx 0.55\times10^{-6}K^{-1}$), a compressive stress might be applied to the suspended area of the ReS_2 flake. It is important to note that the suspended ReS_2 flakes studied here exhibit extremely high aspect ratios, with D/t values ranging from approximately 756 to 1154. Specifically, for a suspended region diameter of D = 5 µm, the aspect ratios correspond to $D/t \approx 1154$ for 5L ($t \approx 4.33$ nm), 1018 for 6L ($t \approx 4.91$ nm), 877 for 7L ($t \approx 5.7$ nm), and 756 for 8L ($t \approx 6.61$ nm). Buckling occurs when the compressive thermal stress in a few-layer ReS_2 flake reaches its critical buckling stress (σ^c) value. To investigate this buckling phenomenon and its potential impact on our measurements, we performed a detailed theoretical analysis. For a simply supported circular flake, the critical buckling stress σ^c can be estimated using Euler's formula,[5]

$$\sigma^c = \frac{2.16^2 E}{12(1-\nu^2)}\left(\frac{t}{R}\right)^2 \tag{S20}$$

where, R = 2.5 µm represents the radius of the suspended region. For our calculation, we used the experimentally characterized Young's moduli of layered ReS_2 along the '*a*' and '*b*'-axes, reported as E_a = 201 GPa and E_b = 127 GPa, respectively, by Xu *et al.*[6] along with a Poisson's ratio $\nu \approx 0.21$. The flake thicknesses used were t ~ 4.33 nm (5L), 4.91 nm (6L), 5.7 nm (7L), 6.61 nm (8L). Based on these parameters, the calculated critical buckling stress σ^c along a-axis (σ_a^c) and b-axis (σ_b^c) are as follows: for five-layer (5L) ReS_2, 256.2 KPa and 154.9 KPa; for six-layer (6L) ReS_2, 329.4 KPa and 199.2 KPa; for seven-layer (7L) ReS_2, 444 KPa and 268.5 KPa; and for eight-layer (8L) ReS_2, 597 KPa and 361.1 KPa, respectively. When the

compressive stress induced by the temperature rise reaches the critical buckling stress along the '*a*'-axis (σ_a^c) and '*b*'-axis (σ_b^c), respectively, the flake undergoes buckling. Beyond this point, the flake cannot sustain additional stress, as it is relaxed through the buckling-induced flake deformation.

The maximum Raman peak shift resulting from compressive thermal stress can be estimated by substituting the critical buckling stress σ_c into Eq. (S12). The stress coefficient along the '*a*'-axis (K^a) and '*b*'-axis (K^b) can be estimated from the strain coefficients of few-layer ReS_2 characterized by Li *et al*.[7] To calculate the stress coefficient, the strain coefficients were first determined from gauge factors, defined as the strain-induced frequency shifts of the corresponding Raman modes: mode III for the '*a*'-axis and mode V for the '*b*'-axis. Subsequently, using Hooke's law, stress coefficients were evaluated, yielding $K^a \sim -0.428$ cm^{-1}/GPa along '*a*'-axis and $K^b \sim -0.56$ cm^{-1}/GPa along '*b*'-axis. The corresponding changes in Raman peak position along mode III and mode V are as follows: for five-layer ReS_2, 10.5×10^{-5} cm^{-1} and 8.5×10^{-5} cm^{-1}; for six-layer ReS_2, 13.5×10^{-5} cm^{-1} and 10.9×10^{-5} cm^{-1}; for seven-layer ReS_2, 18.2×10^{-5} cm^{-1} and 14.7×10^{-5} cm^{-1}; and for eight-layer ReS_2, 24.4×10^{-5} cm^{-1} and 19.8×10^{-5} cm^{-1}, which are much smaller than the typical uncertainty of Raman peak position (~ 0.05 cm^{-1}) in our measurements. For this reason, it is reasonable to assume that the suspended ReS_2 flake remains in a stress-free state.

Luo et al. experimentally demonstrated the no-slip condition by comparing the temperature coefficients of a suspended BTS211 thin film with those of a glass-supported thin film and conducting strain cycle tests.[8] Lee et al. verified the validity of the no-slip condition through their characterization of the elastic properties of graphene.[9] Here, a theoretical analysis of the no-slip condition is presented, followed by a discussion of its experimental validation using our measurements. Under the no-slip condition, the associated change in phonon frequency along the '*a*'-axis and '*b*'-axis can be expressed as,

$$\begin{aligned}\Delta\omega_{no-slip}^{a} &= \left(\frac{K^a\left(\alpha_{sub}-\alpha\right)E_a}{1-\nu}+A^a\right)\Delta T = A_{no-slip}^{a}\Delta T \\ \Delta\omega_{no-slip}^{b} &= \left(\frac{K^b\left(\alpha_{sub}-\alpha\right)E_b}{1-\nu}+A^b\right)\Delta T = A_{no-slip}^{b}\Delta T\end{aligned} \qquad \text{(S21)}$$

where, $\Delta\omega_{no-slip}^{a}$ and $\Delta\omega_{no-slip}^{b}$ denote the shifts in the Raman mode III and mode V peak positions under the no-slip condition, respectively. And, α_{sub} represents the thermal expansion coefficient of an arbitrary substrate, while $A_{no-slip}^{a}$ and $A_{no-slip}^{b}$ denotes the temperature

coefficients of Raman mode III and mode V, respectively, under the no-slip condition on this substrate. However, under the slip condition, the stress acting on the ReS_2 flake is expected to remain nearly constant ($\sigma = \sigma_0$), as any variation in stress is relaxed through interfacial slipping. The associated shifts in the Raman mode III and mode V peak positions under this condition can be expressed as,

$$\begin{aligned} \Delta\omega_{slip}^{a} &= A^{a}\Delta T + K^{a}\sigma_0 = A^{a}\Delta T + \Delta\omega_0^{a} \\ \Delta\omega_{slip}^{b} &= A^{b}\Delta T + K^{b}\sigma_0 = A^{b}\Delta T + \Delta\omega_0^{b} \end{aligned} \tag{S22}$$

where, $\Delta\omega_{slip}^{a}$ and $\Delta\omega_{slip}^{b}$ denote the shifts in the Raman mode III and mode V peak positions under the slip condition, respectively, and $\Delta\omega_0^{a}$ and $\Delta\omega_0^{b}$ are the constant bias along the '*a*'-axis and '*b*'-axis due to the constant stress σ_0. By comparing Eq. (S21) and Eq. (S22), it can be observed that the slope of the phonon frequency response under the no-slip condition ($A_{no-slip}^{a}$ and $A_{no-slip}^{b}$) differs from that under the slip condition (A^{a} and A^{b}) when the thermal expansion coefficient of the substrate α_{sub} is not equal to that of the flake thermal expansion coefficients along the '*a*'-axis (α^{a}) and '*b*'-axis (α^{b}), respectively. This difference in the first-order temperature coefficient has been experimentally confirmed by the temperature-dependent Raman measurements shown in **Figures S12** and **S19** (See the **Table S6** for temperature coefficients) of the Supporting Information, indicating that the no-slip condition was maintained throughout the measurement.

19 Note VI: Verification of uniform stress transfer, negligible initial residual strain, and absence of partial slip in few-layer ReS_2 flakes.

It should be noted that the extraction of the ATEC in this geometry relies on the assumption of an idealized boundary condition specifically, a stress-free initial state and no-slip clamping at the interface between the suspended and supported regions. To validate these assumptions experimentally, we compared the Raman spectra of both the suspended and substrate-supported ReS_2 regions(*7L* and *8L*).

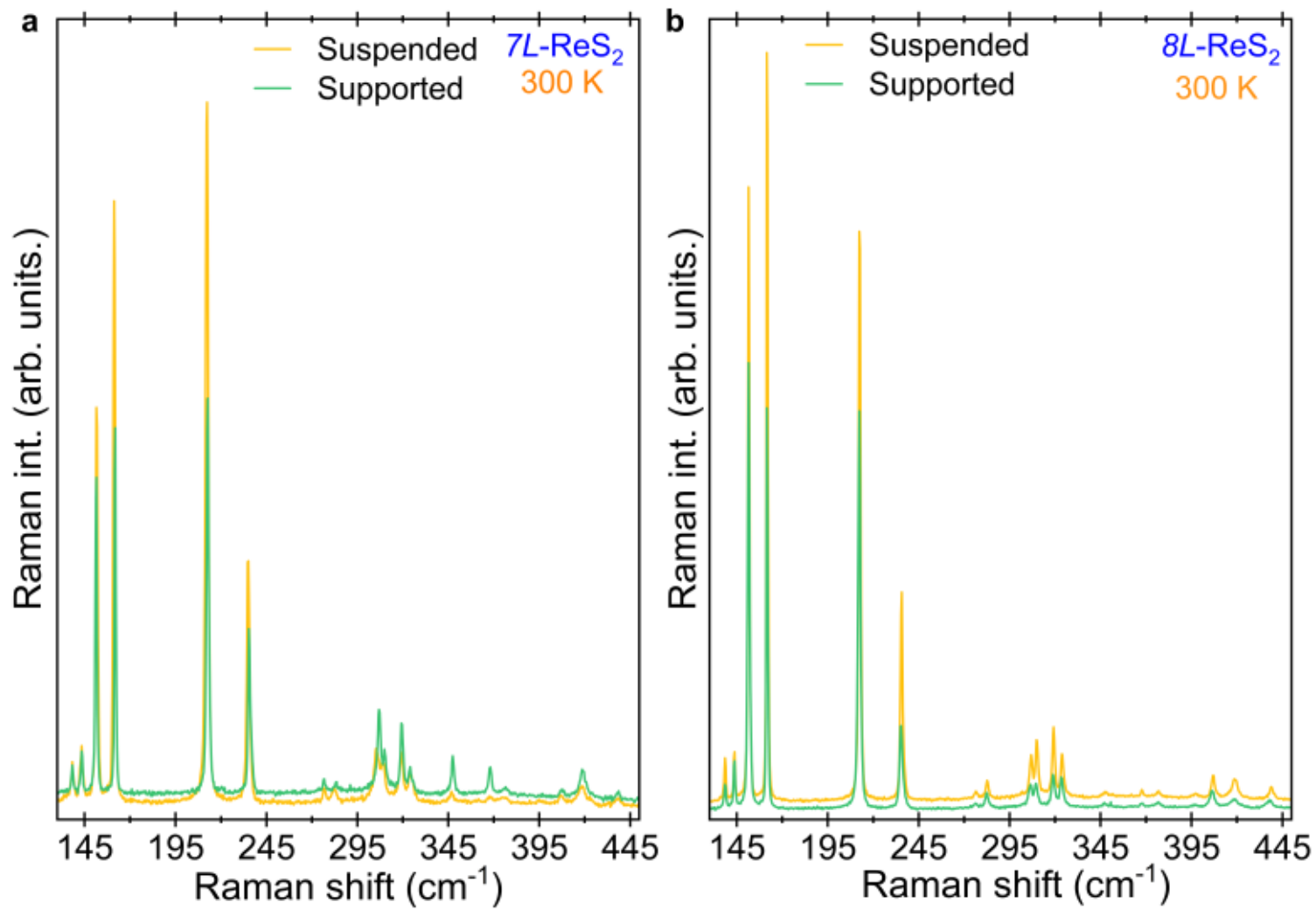

Figure S17. Raman spectra comparing the suspended and supported configurations for (a) *7L* and (b) *8L* ReS_2 flakes.

Although the Raman mode intensities varied due to substrate effects, we observed no significant relative shifts in the mode positions between the two regions at room temperature, nor did we detect any anomalous strain-induced splitting or non-linear shift (See **Fig. S12** and **S19**) at elevated temperatures. Given the strong dependence of Raman mode frequencies on lattice strain, the observed spectral uniformity indicates that initial residual strain is negligible and that the supported ReS_2 remains effectively clamped during thermal expansion, without undergoing macroscopic partial slip. Nevertheless, standard dry-transfer fabrication inherently leaves trace interfacial residues that can cause heterogeneous adhesion at the microscopic level. While our Raman data confirms these effects do not induce macroscopic strain gradients, we cautiously frame the extracted ATEC values as effective parameters characteristic of the physical device architecture.

20 Temperature-dependent Raman study on substrate-supported few-layer ReS_2

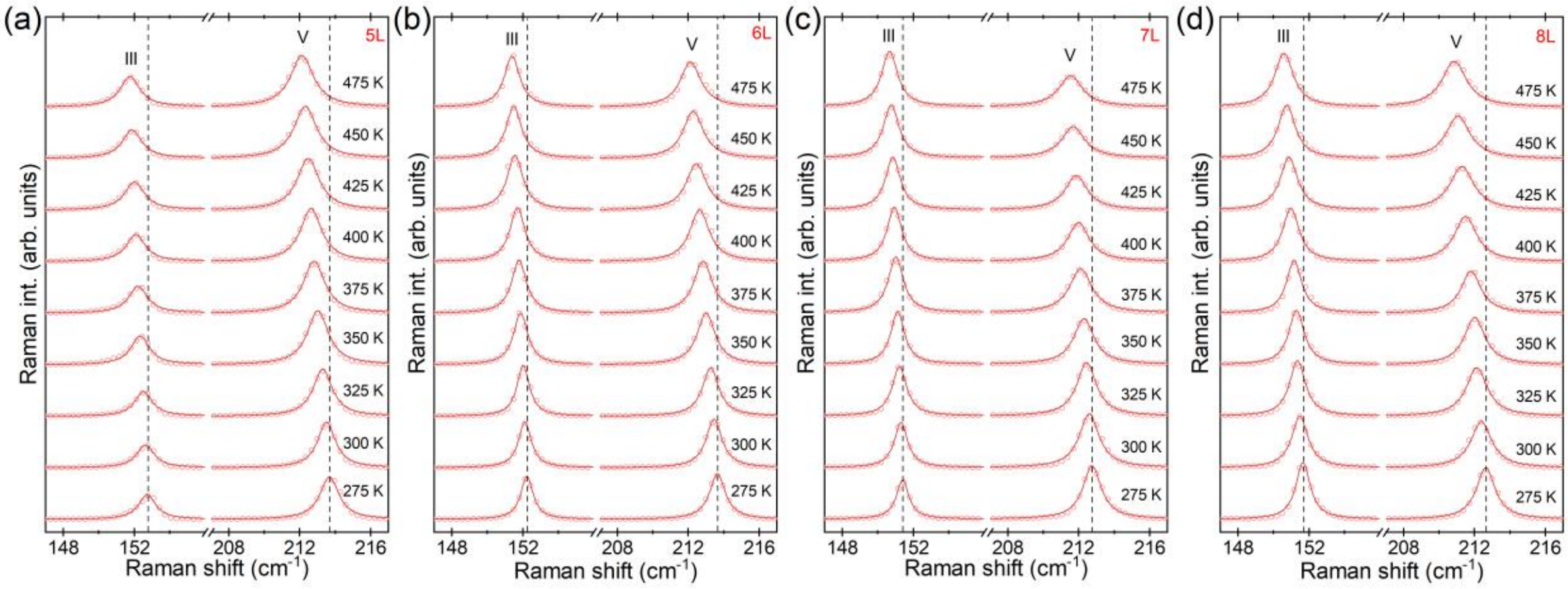

Figure S18. Raman spectra of substrate-supported few-layer ReS_2 at different temperatures. (a-d) Raman spectra of the *5L*, *6L*, *7L*, and *8L* substrate-supported ReS_2 flakes were measured over the temperature range 275 K to 475 K, respectively. The black dotted lines indicate the reference peak positions of modes III and V at 275 K.

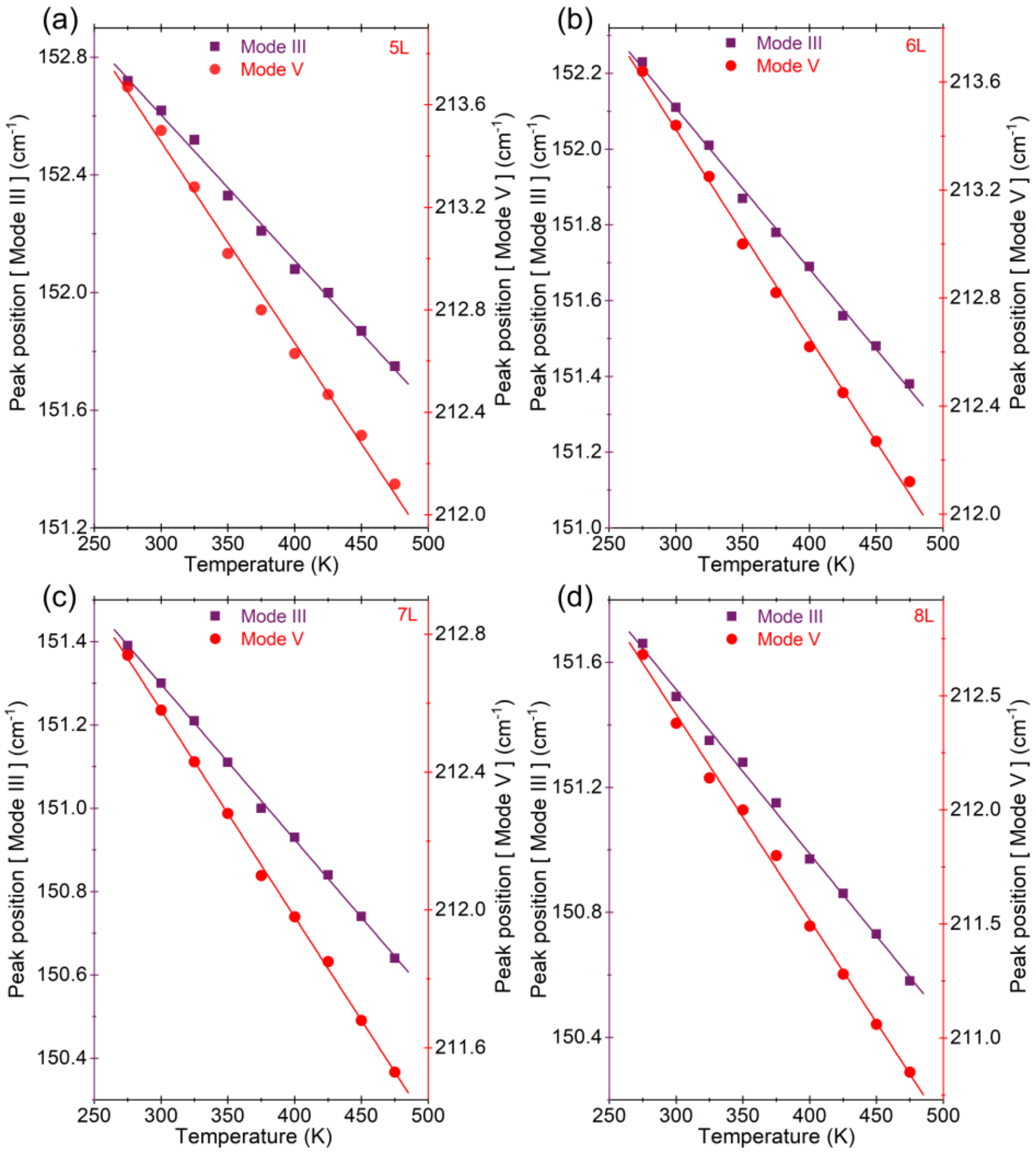

Figure S19. Peak positions of Raman modes (III and V) as a function of temperature. (a-d) Temperature-dependent shifts in the peak positions of Raman modes III (violet rectangles) and V (red circles) in suspended five-layer (*5L*), six-layer (*6L*), seven-layer (*7L*), and eight-layer (*8L*) ReS_2 flakes, respectively.

21 Table S6: Layer-dependent temperature coefficients of few-layer suspended ($\chi_T^{Suspended}$) and substrate-supported ($\chi_T^{Supported}$) ReS_2 along the '*a*' and '*b*'-axes

Number of layers (nL)	$\chi_T^{Suspended}$ [$10^{-2} \times cm^{-1}K^{-1}$]		$\chi_T^{Supported}$ [$10^{-2} \times cm^{-1}K^{-1}$]	
	'*a*'-axis	'*b*'-axis	'*a*'-axis	'*b*'-axis
5L	-0.533±0.011	-0.835±0.014	-0.495±0.012	-0.785±0.016
6L	-0.455±0.009	-0.815±0.015	-0.425±0.008	-0.771±0.014
7L	-0.393±0.006	-0.634±0.005	-0.373±0.004	-0.600±0.007
8L	-0.542±0.010	-0.931±0.016	-0.526±0.012	-0.901±0.018

22 Table S7: Layer-dependent in-plane thermal expansion coefficients of ReS_2 along the '*a*' and '*b*'-axes, together with their anisotropy ratios

Layer Number (nL)	Thermal Expansion Coefficient along '*a*'-axis $10^{-6} \times K^{-1}$	Thermal Expansion Coefficient along '*b*'-axis $10^{-6} \times K^{-1}$	Anisotropic Ratio
5L	4.04 ± 0.1	6.10 ± 0.22	1.51
6L	3.30 ± 0.1	5.44 ± 0.11	1.65
7L	2.38 ± 0.19	4.33 ± 0.23	1.82
8L	2.02 ± 0.19	3.88 ± 0.23	1.92

References:

[1] J. Menéndez, M. Cardona, *Phys. Rev. B* **1984**, *29*, 2051.

[2] Mildred S. Dresselhaus, *Group theory: application to condensed matter physics*, **2010**.

[3] Z. Luo, J. Maassen, Y. Deng, Y. Du, R. P. Garrelts, M. S. Lundstrom, P. D. Ye, X. Xu, *Nat. Commun.* **2015**, *6*, 1.

[4] W. Bao, F. Miao, Z. Chen, H. Zhang, W. Jang, C. Dames, C. N. Lau, *Nat. Nanotechnol.* **2009**, *4*, 562.

[5] T. C. Lim, *J. Eng. Mater. Technol.* **2014**, *136*, 1.

[6] B. Xu, J. Zhu, F. Xiao, C. Jiao, Y. Liang, T. Wen, S. Wu, Z. Zhang, L. Lin, S. Pei, H. Jia, Y. Chen, Z. Ren, X. Wei, W. Huang, J. Xia, Z. Wang, *Small* **2023**, *19*, 1.

[7] H. Li, D. Y. Lin, A. Di Renzo, S. Puebla, R. Frisenda, X. Gan, J. Quereda, Y. Xie, A. M. Al-Enizi, A. Nafady, A. Castellanos-Gomez, *Appl. Phys. Lett.* **2022**, *120*.

[8] R. Yan, J. R. Simpson, S. Bertolazzi, J. Brivio, M. Watson, X. Wu, A. Kis, T. Luo, A. R. Hight Walker, H. G. Xing, **2013**.

[9] C. Lee, X. Wei, J. W. Kysar, J. Hone, *Science (80).* **2008**, *321*, 385.
